# Late blight resistance gene from *Solanum ruiz-ceballosii *is located on potato chromosome X and linked to violet flower colour

**DOI:** 10.1186/1471-2156-13-11

**Published:** 2012-02-27

**Authors:** Jadwiga Śliwka, Henryka Jakuczun, Marcin Chmielarz, Agnieszka Hara-Skrzypiec, Iga Tomczyńska, Andrzej Kilian, Ewa Zimnoch-Guzowska

**Affiliations:** 1 Plant Breeding and Acclimatization Institute-National Research Institute, Młochów Research Centre, Platanowa 19, 05-831 Młochów, Poland; 2 Diversity Arrays Technology Pty Ltd., 1 Wilf Crane Cr., Yarralumla, ACT 2600, Australia

**Keywords:** Diversity Array Technology, Mapping, *Phytophthora infestans*, *Solanum tuberosum*, *Rpi-rzc1*

## Abstract

**Background:**

*Phytophthora infestans *(Mont.) de Bary, the causal organism of late blight, is economically the most important pathogen of potato and resistance against it has been one of the primary goals of potato breeding. Some potentially durable, broad-spectrum resistance genes against this disease have been described recently. However, to obtain durable resistance in potato cultivars more genes are needed to be identified to realize strategies such as gene pyramiding or use of genotype mixtures based on diverse genes.

**Results:**

A major resistance gene, *Rpi-rzc1*, against *P. infestans *originating from *Solanum ruiz-ceballosii *was mapped to potato chromosome X using Diversity Array Technology (DArT) and sequence-specific PCR markers. The gene provided high level of resistance in both detached leaflet and tuber slice tests. It was linked, at a distance of 3.4 cM, to violet flower colour most likely controlled by the previously described *F *locus. The marker-trait association with the closest marker, violet flower colour, explained 87.1% and 85.7% of variance, respectively, for mean detached leaflet and tuber slice resistance. A genetic linkage map that consisted of 1,603 DArT markers and 48 reference sequence-specific PCR markers of known chromosomal localization with a total map length of 1204.8 cM was constructed.

**Conclusions:**

The *Rpi-rzc1 *gene described here can be used for breeding potatoes resistant to *P. infestans *and the breeding process can be expedited using the molecular markers and the phenotypic marker, violet flower colour, identified in this study. Knowledge of the chromosomal localization of *Rpi-rzc1 *can be useful for design of gene pyramids. The genetic linkage map constructed in this study contained 1,149 newly mapped DArT markers and will be a valuable resource for future mapping projects using this technology in the *Solanum *genus.

## Background

Domestication of crop plants involving selection through many generations has brought yield and quality improvement but also narrowed the gene pools of cultivated species [1]. Therefore, for the last hundred years, modern plant breeders have searched wild relatives of crop plants for valuable genes, alleles and Quantitative Trait Loci (QTL) encoding desirable phenotypic characters. In potato, more than 210 wild and cultivated species [1] provide a good chance of finding useful traits. However, till date only a limited number has been used in breeding programs. Exploitation of wild *Solanum *species has focused on those resistant to pests and pathogens. Among potato pathogens, *Phytophthora infestans *(Mont.) de Bary, the causal organism of late blight, is economically the most important. Resistance against it has been one of the primary goals of potato breeding [2]. The first introduction of major genes for resistance (R genes) to *P. infestans *from the wild species *S. demissum *early in the 20th century brought disappointment, due to the rapid spread of *P. infestans *strains possessing the corresponding virulence factors. Cultivars with different combinations of these genes quickly became infected [3]. However, some potentially more durable, broad-spectrum R genes have recently been described, including *RB/Rpi-blb1 *[4,5], *Rpi-blb3 *[6], *Rpi-vnt1.1 *[7,8], *Rpi-phu1 *[9] and *Rpi-sto1 *[10]. New strategies preventing such rapid evolution of compatible *P. infestans *races have been proposed. All these are based on avoiding monocultures and use of as many broad-spectrum R genes as possible. These can either be stacked within cultivars or used in mixtures of breeding lines each containing a different R gene. More simply, cultivars containing different R genes could be grown next to each other or could be changed between seasons to achieve R gene diversification [11]. Use of molecular markers and transgenesis can facilitate this process. Triple R gene transformants have been obtained stacked with three R genes against *P. infestans*, *Rpi-sto1*, *Rpi-vnt1.1 *and *Rpi-blb3*, and these plants combined resistance spectra of the individual R genes [12]. PCR markers have also been applied in selection of plants possessing both *R_pi-mcd1 _*gene from *S. microdontum *[13] and *R_Pi-ber _*from *S. berthaultii *[14-16], though these two genes provided only limited resistance [17]. To improve the likelihood that these strategies will be successful, breeders need to have many such genes available and therefore the search for these continues among wild relatives of potato.

*Solanum ruiz-ceballosii *Cárd. (syn. *Solanum sparsipilum *(Bitt.) Juz. et Buk.) is a species closely related to potato, diploid, 2 EBN (Endosperm Balance Number), originating from Peru and Bolivia. It is a polymorphic weed species of walls, fields and field borders growing at altitudes of 2,400-3,800 m, with a corolla pale to medium blue-violet [18]. It is also known as *Solanum ruiz-zeballosii *Cárd. and, together with *S. sparsipilum *(Bitt.) Juz. et Buk., is accepted as belonging to the species *Solanum brevicaule *Bitt. [19] or is included in the brevicaule-complex [20]. The distribution of *S. brevicaule *is similar to that of the species mentioned above, spanning Bolivia near the border with Peru and south to northwest Argentina, from 2,000 up to 4,180 m above sea level and its corolla colour is described as purple violet to light blue [19]. Another slightly differently spelled name, *Solanum ruiz-cevallosii *Cárd., was published together with the proposed species abbreviation *rzc *[21]. Being a diploid, 2 EBN species, *S. ruiz-ceballosii *can be crossed with diploid *S. tuberosum*, but seeds were also obtained from its cross with a tetraploid potato cultivar Aurora [22].

*S. ruiz-ceballosii *was previously described as highly resistant to potato late blight both in leaves and in tubers [23,24]. Quantitative trait locus for late blight resistance with major effects originating from the synonymous *S. sparsipilum *has previously been mapped on potato chromosome X using stem and foliage tests and explained up to 29% of variance in the stem resistance of the mapping population [25]. *S. sparsipilum *has been also described as a source of resistance to the nematodes *Meloidogyne fallax *[26] and *Globodera pallida *[27] as well as to Potato Virus Y (PVY) [28]. Therefore, it may become a valuable component of potato breeding programs. In the case of PVY, a dominant resistance gene *Ncspl *originating from *S. sparsipilum *has been mapped to potato chromosome IV [28]. In addition, *S. sparsipilum *has been proposed as a potential source of resistance against potato tuber moth, *Phthorimaea operculella *[29].

The goal of this study was to characterize the late blight resistance of the clone chosen from *S*. *ruiz-ceballosii *accession VIR 8664 (VIR 7370) [23,24], originating from the Vavilov Collection in Russia (VIR), and to map the underlying R gene, named *Rpi-rzc1*. Within our study, the *Rpi-rzc1 *gene was mapped to potato chromosome X, it proved to be effective in providing a high level of resistance to *P. infestans *both in detached leaflet and in tuber slice tests. We also noted a close linkage between *Rpi-rzc1 *and violet flower colour encoded by the *F *locus [30,31], which may be useful for selection of resistant individuals from certain crosses.

## Methods

### Plant material

A late blight resistant clone 99-10/36, selected from *S. ruiz-ceballosii *accession VIR 7370 (VIR 8664) obtained from the VIR collection in Russia and collected by Cárdenas in Horlahon, Bolivia, and a susceptible *S. tuberosum *dihaploid of the Polish cultivar (cv.) Balbina (dH Balbina) from IHAR-PIB O/Młochów's haploidization program, were crossed to obtain an F_1 _mapping population of 114 individuals. The parent dH Balbina and the progeny were propagated each year in the field, whereas clone 99-10/36 was propagated in the glasshouse. Along with the mapping population and the parental clones, standard cultivars (cvs) proposed by the Eucablight consortium [32], Alpha, Bintje (susceptible to late blight), Biogold (moderately resistant, containing an R gene), Eersteling (susceptible), Escort (resistant, with *R1, R2, R3 *and *R10 *[33]), Gloria, Robijn (moderately resistant), Sárpo Mira (very resistant) and two additional diploid hybrid clones, DG 94-15 (resistant in leaflets and tubers) and DG 94-668 (resistant in tubers) [9], were included in tests of resistance to *P. infestans*. From the mapping population, 13 highly resistant individuals were chosen on the basis of the laboratory tests results. These individuals were assessed for resistance in the field conditions. Standards, except cv. Biogold and diploid clones, were also included in the field test.

### *P. infestans* isolate

The isolate MP324 from the pathogen collection of the Plant Breeding and Acclimatization Institute-National Research Institute, Młochów, Poland was used in all resistance tests. The isolate, collected in 1997 in Poland, was of A1 mating type, highly aggressive, metalaxyl resistant and of complex race (1.2.3.4.5.6.7.8.10.11). Black's differential set, obtained from the Scottish Agricultural Science Agency, Edinburgh, UK, was used to confirm the isolate's virulence each time in parallel to the tests. Before each resistance test, the isolate was passaged through susceptible potato tissue at least twice. Isolate MP324 has frequently been used in resistance tests in our laboratory [9,34].

### Late blight resistance assessment

The resistance to *P. infestans *of the parental clones, the mapping population and the standard cultivars was evaluated by laboratory detached leaflet and tuber slice tests as described earlier [9,34]. Detached leaflets or tuber slices were inoculated with a 30 μl droplet of sporangia/zoospore suspension (50 sporangia/μl). After incubation of 6 days at 16°C, in high humidity and, in case of leaflets, under constant light of about 1,600 lx, they were scored using a 1-9 scale, where 9 is maximum resistant [9,34]. The detached leaflet tests were replicated as follows: 3 leaflets/genotype × 2 replications × 2 dates were tested in 2007, 3 leaflets/genotype × 2 replications in 2008 and 3 leaflets/genotype × 2 replications × 2 dates in 2010. Three tuber slices/genotype × 2 replications were tested in 2006, 2007 and 2010. A genotype was considered resistant, i.e. possessing the R gene, when its mean resistance score in both detached leaflet and in tuber slice tests was ≥ 7.

In 2008 and 2009 field resistance assessment of 13 individuals from the mapping population and seven standards (Table 1) was conducted at Southeast Poland in Boguchwala, where the weather conditions were favorable for severe late blight infection. Material was planted in randomized 4-hill plots, in two replications in 2008 and in one replication in 2009. Natural infection was evaluated weekly from the first symptoms of pathogen. Values of rAUDPC (area under disease progress curve) were calculated from 6 to 8 readings of infection in scale 1-9, according Fry (1978) [35].

**Table 1:** Mean rAUDPC values of field infection for 13 individuals and standard cultivars

Genotype	rAUDPC in	
	**2008**	**2009**

05-18/1	0.012	0.000
05-18/9	0.000	0.000
05-18/24	0.000	0.000
05-18/25	0.000	0.000
05-18/32	0.000	0.001
05-18/33	0.000	0.001
05-18/54	0.000	0.000
05-18/56	0.000	0.000
05-18/73	0.000	0.000
05-18/98	0.000	0.000
05-18/99	0.000	0.000
05-18/118	0.000	0.001
05-18/129	0.000	0.000

standard cultivars		

Alpha	0.425	0.312
Bintje	0.532	0.791
Gloria	0.512	0.722
Eerstling	0.446	0.590
Escort	0.371	0.426
Robijn	0.307	0.273
Sárpo Mira	0.029	0.000

**Figure 1:**
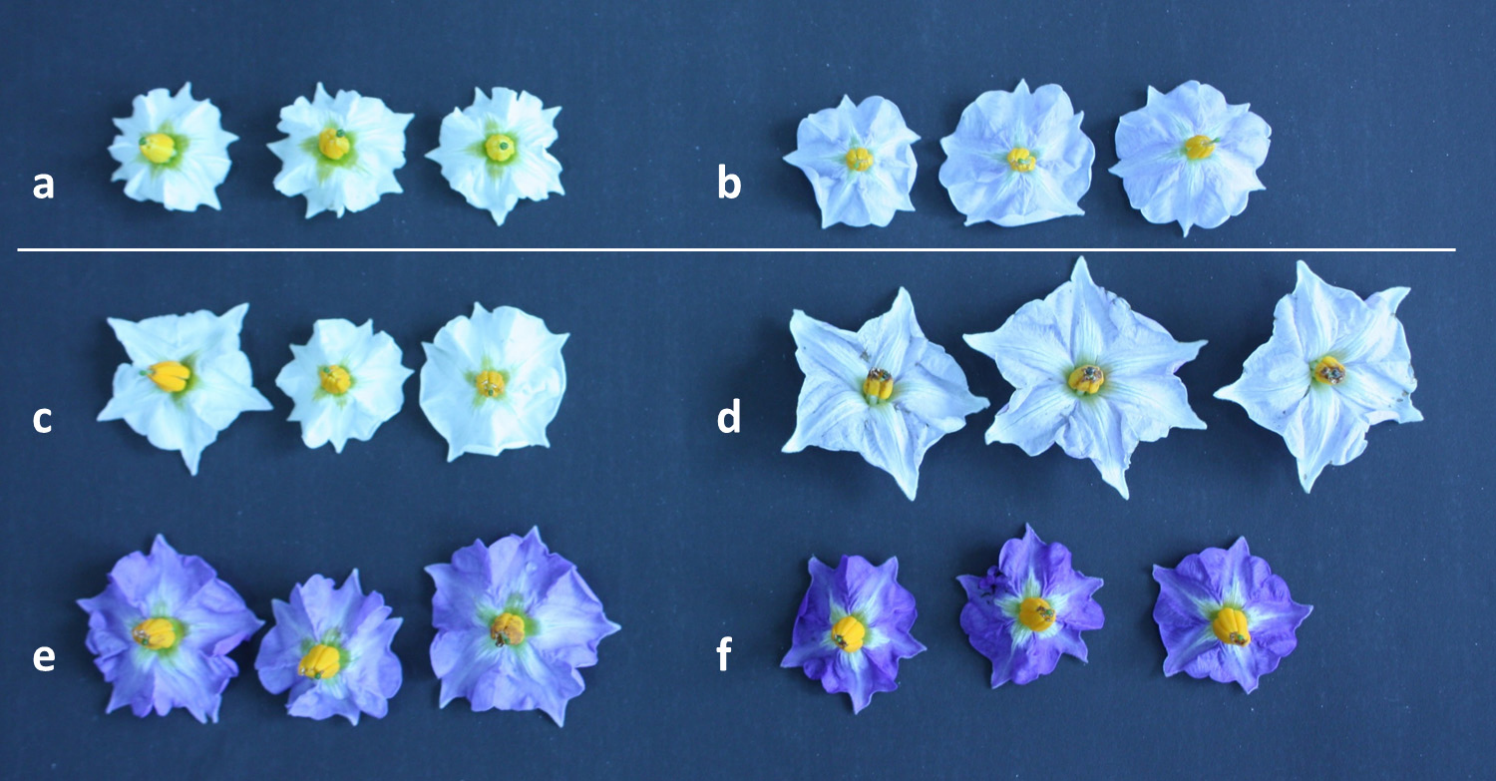
**Flower colour of the parents dH Balbina (a - white) × *S. ruiz-ceballosii *99-10/36 (b - pale violet) and examples of the flower colours of F_1 _individuals: c -white; d - pale violet; e - violet; f - dark violet**.

*Flower colour *was assessed visually; four categories were noted in the mapping population dH Balbina × 99-10/36: (1) white, (2) pale violet, (3) violet and (4) dark violet corolla. The flower colours of the parents and examples from the progeny are shown in Figure 1. Categories 2-4 were included into the general violet group for the purpose of mapping. Assessments were repeated in 2006, 2007 and 2008, but not all individuals flowered each year.

### DNA isolation and sequence-specific markers

Genomic DNA was extracted from 1 g of fresh, young leaves of field grown plants with the DNeasy Plant Maxi kit (Qiagen, Hilden, Germany). All sequence-specific markers listed in Additional file 1 were amplified using the following conditions as described by Śliwka et al. (2011) [36]. The reaction mixture of 20 μl contained 2 μl of 10 × PCR buffer, the four deoxynucleotides (0.1 mM), MgCl_2 _(1.5 mM), primers (0.2 μM), *Taq *polymerase (0.05 U/μl) and 10-30 ng of template DNA. The PCR program was 94°C - 180 s; 40 cycles of: 94°C - 30 s, 55°C - 45 s, 72°C - 90 s; 72°C - 420 s; where the annealing temperature was modified depending on primers used, this is indicated in Additional file 1. The reactions of the PCR products with the corresponding restriction endonucleases, also listed in Additional file 1 were performed according to the manufacturer's recommendations.

### Diversity Array Technology (DArT)

The DArT analysis was performed in Diversity Array Pty Ltd. Canberra, Australia, exactly as described for *S. michoacanum *[36], following the protocols previously developed for other plant species [37-40].

### Statistical and linkage analyses

The normality of the distribution of the phenotypic data was checked by the Kolmogorov-Smirnov test. The reproducibility of the resistance tests between years as well as the correlations between traits were evaluated by calculating the linear Pearson's correlation coefficients. Marker--trait linkages and determination coefficients (*R^2^*) were estimated by the Student's *t*-test and analysis of variance, respectively. The fit of segregation to the expected ratio was checked by the *χ*^2 ^test. All statistical analyses were performed using computer program STATISTICA for Windows (Stat Soft, Inc., Tulsa, OK, U.S.A.). Linkage analyses were performed using JoinMap ^® ^4 [41] with the following settings: CP population type (creating maternal and paternal linkage maps first and then creating a common population map), independence LOD as a grouping parameter (linkages with LOD > 3 were considered significant), regression mapping algorithm and Haldane's mapping function.

## Results

### Late blight resistance assessment

**Figure 2:**
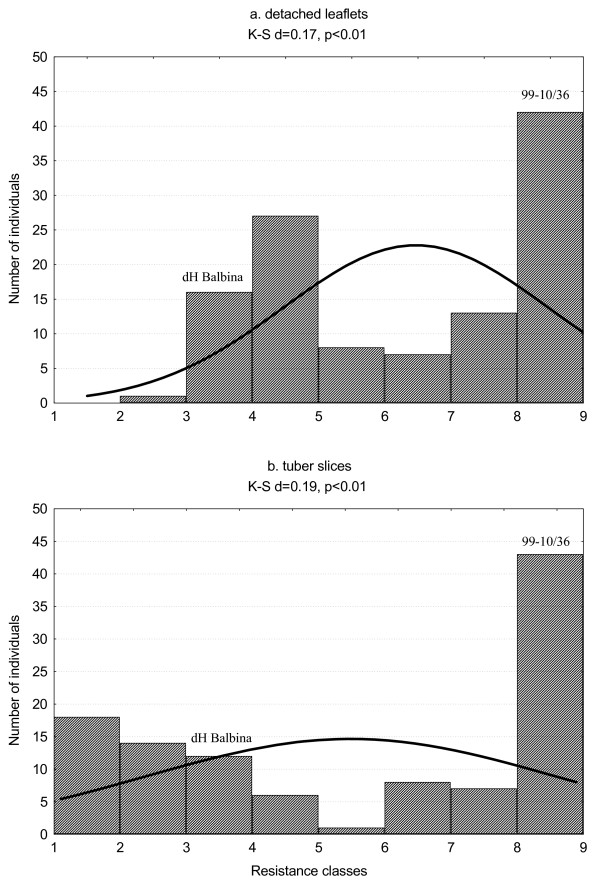
**Distributions of (a) mean (2007, 2008 and 2010) leaflet resistance to *P. infestans *and (b) mean (2006, 2007 and 2010) tuber slice resistance in the mapping population**. The fitness to the normal curve: K-S - Kolmogorov-Smirnov test, d - coefficient calculated for this test, p - probability, the line indicates the normal curve. Resistance levels of parental clones are marked with their names: 99-10/36 and dH Balbina.

The mean resistance scores of the parental clones 99-10/36 and dH Balbina as well as of the resistance standards are presented in Table 2*S. ruiz-ceballosii *clone 99-10/36 was the most resistant in both detached leaflet and tuber slice tests, with mean scores 9.0 and 8.9, respectively. Most of the standards performed as expected with cv. Sárpo Mira being highly resistant and cvs Biogold and Escort and clone DG 94-15 being moderately resistant in detached leaflet tests. Cvs Gloria and Robijn and clone DG 94-668 proved more susceptible than expected. In tuber slice tests, cvs Sárpo Mira and Robijn and clone DG 94-15 showed some level of resistance to *P*. *infestans *(Table 2). The mean detached leaflet resistance results obtained for the mapping population in three subsequent years of testing were correlated with each other, with Pearson's correlation coefficients r = 0.581 (2007-2008), r = 0.664(2007-2010) and r = 0.783 (2008-2010) (p < 0.000 in all cases). Similarly, the tuber slice resistance results were correlated between years with Pearson's correlation coefficients r = 0.681 (2006-2007), r = 0.767 (2006-2010) and r = 0.742 (2007-2010); p < 0.000 in all cases. There was also a strong correlation between the mean (2007-2010) detached leaflet resistance results and the mean (2006-2010) tuber slice resistance results (Pearson's correlation coefficient r = 0.862, p < 0.000), indicating that the same genetic factor(s) was effective in both foliage and tuber resistance to *P. infestans *in the mapping population. Analysis of variance showed that plant genotype had the most significant effect on the resistance test results both in detached leaflet tests and in tuber slice tests. There were also significant effects of the interaction genotype × year of testing and of year of testing alone (Table 3). The distributions of the mean detached leaflet and tuber slice resistances in the mapping population were bimodal and significantly deviated from normality, which was confirmed by the Kolmogorov-Smirnov test (Figure 2). The range of the mean detached leaflet scores was 3.0-9.0 which was narrower than the case of tuber slice tests where the results ranged from 1.0 to 9.0 and covered the full assessment scale. The mean resistance ratings for the population were 6.5 and 5.5 in the leaflet and tuber slice tests, respectively, indicating less resistance in the tubers or stronger infection pressure in the tuber slice tests. When the individuals of the mapping population with mean resistance ≥ 7 were included into the resistant class, the sizes of the resistant and susceptible classes did not significantly deviate from the 1: 1 ratio expected for segregation of a single gene. This applied to the results of both the detached leaflet tests, where 52 individuals were assessed as resistant and 62 as susceptible (*χ*^2 ^= 0.89, df = 1, p < 0.35), and the tuber slice tests where the resistant: susceptible ratio was 51: 63 (*χ*^2 ^= 1.26, df = 1, p < 0.26). Only in 16 cases, was the leaflet resistance of a specific individual slightly above the threshold while the tuber resistance was below it, or vice versa, which we interpret as being due to test variation rather than genuine genetic difference. The majority of the individuals (98 out of 114) were either resistant both in detached leaflet and tuber slice tests or susceptible in both types of tests.

**Table 2:** The late blight resistance of parental clones and standards in (a) detached leaflet and (b) tuber slice tests.

	a. Resistance of detached leaflets					b. Resistance of tuber slices				
	
Genotype	2007	2008	2010	Weighted mean: 2007, 2008 and 2010	SD	2006	2007	2010	Mean: 2006, 2007 and 2010	SD
99-10/36	9.0	9.0	9.0	**9.0**	**0.0**	9.0	8.6	9.0	**8.9**	**0.2**
dH Balbina	3.8	4.7	3.4	**3.8**	**0.7**	4.3	2.5	4.3	**3.7**	**1.0**
Alpha	2.7	4.5	5.2	**4.1**	**1.3**	2.0	3.55	4.1	**3.2**	**1.1**
Bintje	2.6	5.1	4.2	**3.7**	**1.3**	2.1	2	2.1	**2.1**	**0.1**
Biogold	6.5	8.9	6.8	**7.1**	**1.3**	4.2	1.75	4.1	**3.4**	**1.4**
Eersteling	2.0	2.9	2.7	**2.5**	**0.5**	1.8	5.5	2.5	**3.3**	**2.0**
Escort	4.7	8.0	5.0	**5.5**	**1.8**	2.9	3.5	2.3	**2.9**	**0.6**
Gloria	2.8	5.5	2.9	**3.4**	**1.5**	1.3	4.2	1.6	**2.4**	**1.6**
Robijn	3.1	6.1	3.5	**3.9**	**1.6**	4.0	7.15	3.3	**4.8**	**2.1**
Sárpo Mira	8.8	9.0	8.8	**8.8**	**0.1**	5.9	7.35	4.7	**6.0**	**1.3**
DG 94-15	6.1	6.9	6.4	**6.4**	**0.4**	7.1	7.25	2.9	**5.8**	**2.5**
DG 94 -668	2.8	4.6	2.8	**3.2**	**1.0**	4.6	3.55	5.0	**4.4**	**0.7**

In the case of the detached leaflet tests, a weighted mean was calculated, since the numbers of tested leaflets per genotype were not equal in all years

*SD *standard deviation

**Table 3:** Analysis of variance calculated on mean scores of each testing date in (a) detached leaflet tests across three years and (b) in tuber slice tests done in 2006, 2007 and 2010 in the mapping population dH Balbina × 99-10/36

Factor	Df^a ^effect	Mean sum of squares effect	Df^a ^error	Mean sum of squares error	F	P	R^2 ^(%)^b^
a. detached leaflet tests							

{1}year	2	79.2	137	0.4	194.92	0.000	4.82
{2} genotype	115	20.8	137	0.4	51.26	0.000	72.87
Interaction: 1 × 2	162	3.9	137	0.4	9.53	0.000	19.09

b. tuber slice tests							

{1}year	2	55.1	137	1.2	44.21	0.000	1.71
{2} genotype	115	43.8	137	1.2	35.19	0.000	78.30
Interaction: 1 × 2	162	5.5	137	1.2	4.43	0.000	13.88

^a ^- number of degrees of freedom. ^b ^- percentage of variance explained

Based on field observations of late blight infection of highly resistant individuals selected from mapping population values of rAUDPC were calculated (Table 1). In two years all 13 tested individuals showed high field resistance to *P. infestans*. Mapping data confirmed the presence of *Rpi-rzc1 *gene in these individuals. Standard cvs, beside resistant cv. Sárpo Mira, were strongly infected, rAUDPC values of the most susceptible standard, cv. Bintje were 0.532 and 0.791, in 2008 and 2009, respectively.

### Flower colour assessment

White flowers of the dH Balbina and pale violet flowers of the *S. ruiz-ceballosii *99-10/36, as well as examples of the four flower colour categories noted in the progeny are shown in the Figure 1. The flower colour of 11 progeny individuals could not be assessed unambiguously due to colour discrepancies between the three years of assessment. Lack of flowering, flower fading and non-homogenous genotype could be responsible for this. Of the remaining 103 individuals, 51 had violet flowers in three different shades of violet (Figure 1) and 52 had white flowers, which is a 1: 1 segregation as confirmed by the *χ*^2 ^test (*χ*^2 ^= 0.01, df = 1, p < 0.92). Violet flower colour was strongly correlated with higher mean leaflet (Pearson's correlation coefficient r = 0.882, p < 0.000) and tuber resistance (Pearson's correlation coefficient r = 0.804, p < 0.000).

### Linkage map

Firstly two parental linkage maps and then the common CP map were constructed using the JoinMap ^® ^4 software. The common map consisted of 1603 DArT markers and 48 reference sequence-specific PCR markers of known chromosomal localization (Table 4 Additional file 2). Using those PCR markers (Additional file 1 Additional file 2) all 12 potato chromosomes could be identified, although one marker, C2_At1g05055 [42], was mapped to chromosome VI, that is a different position than the expected one on chromosome IV. On average, 134 markers were located on a chromosome and this number ranged from 74 on chromosome IV to 195 on chromosome V. Total map length reached 1204.8 cM and the particular chromosomes varied in length from 79.1 cM (chromosome X) to 143.2 cm (chromosome III), with an average length of 100.4 cM. The mean interval between markers was 0.75 cM, although the markers were not distributed evenly.

**Table 4:** Linkage map of the F_1 _population dH Balbina × *S.ruiz-ceballosii *99-10/36 and its comparison to the reference *Solanum phureja *diploid map 2010 [42] and *S. michoacanum *map [36]

Chromo-some	Number of markers	Number of reference markers	Length (cM)	Number of markers common with *S. phureja *map:		Number of markers common with *S. michoacanum *map:	
				**concordant position**	**discordant position**	**concordant position**	**discordant position**

I	167	4	103.5	36	1	13	2
II	168	4	113.9	41	1	26	-
III	183	4	143.2	44	5	2	-
IV	74	2	93.9	21	3	6	1
V	195	3	91.8	36	2	18	1
VI	125	3 + 1^a^	134.8	11	-	9	1
VII	123	5	82.3	18	-	2	2
VIII	186	5	90.9	53	1	16	1
IX	84	4	107.0	24	4	1	-
X	75	4	79.1	14	5	7	1
XI	124	4	81.8	29	5	7	5
XII	99	5	82.6	5	2	15	1
**Total**	1,603	47 + 1^a^	1204.8	332	29	122	15

^a ^marker that on Tomato-EXPEN 2,000 map was located on chromosome IV [42]

### The *Rpi-rzc1* gene and the flower colour locus

**Figure 3:**
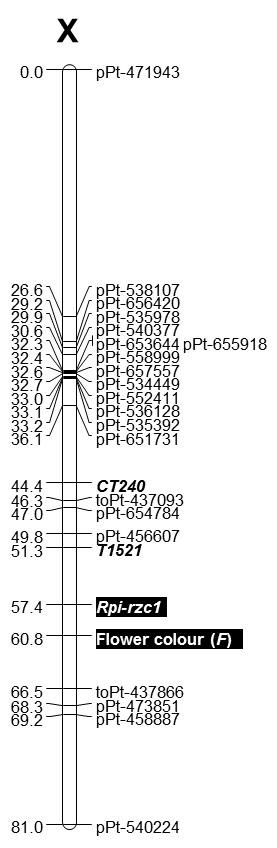
**Genetic linkage map of the *S. ruiz-ceballosii *99-10/36 chromosome X showing the location of the late blight resistance gene and the flower colour locus marked by black rectangles**. The reference PCR markers are in bold italics. On the left, cumulative genetic distances in cM are given.

The *Rpi-rzc1 *gene for resistance to potato late blight was mapped to chromosome X of the resistant parent, *S. ruiz-ceballosii *99-10/36 (Figure 3). The gene was linked to the flower colour locus with a distance of 3.4 cM, and therefore violet flower colour could be treated as an additional, phenotypic marker for the presence of the *Rpi-rzc1 *gene. Nine markers showed statistically significant linkage with both detached leaflet and tuber slice resistance to *P*. *infestans *in each year of testing as confirmed by T-tests (Table 5). The marker-trait association with the closest marker, that is violet flower colour, explained 87.1% and 85.7% of variance, respectively in mean detached leaflet and tuber slice resistance. While, as expected, distal to the *Rpi-rzc1 *gene the percentages of the variance explained by the marker-trait associations decreased as the distance from the gene increased, proximal to the gene they seemed to fluctuate (Figure 3, Table 5). This could be explained by the combination of missing data both in marker and resistance scores.

**Table 5:** The percentages of variance (R^2 ^) in *P.infestans *resistance explained by the significant marker-trait linkages (T student test, p < 0.000) in (a) detached leaflet tests and (b) tuber slice tests.

Marker	*R^2 ^*(%)							
	
	a. Resistance of detached leaflets				b. Resistance of tuber slices			
	
	2007	2008	2010	Weighted mean: 2007, 2008 and 2010	2006	2007	2010	Mean: 2006, 2007 and 2010
CT240	58.1	43.0	41.3	54.2	51.0	28.4	36.9	47.2
toPt-437093	53.3	35.3	34.8	46.4	44.8	33.6	32.5	44.8
pPt-654784	69.6	62.3	57.7	73.9	66.9	46.0	54.1	67.8
pPt456607	81.3	49.4	45.0	63.9	63.9	39.6	44.1	59.8
T1521	69.0	43.6	39.8	56.0	52.1	31.1	36.8	48.6
Violet flower colour	70.9	74.7	75.9	87.1	76.9	61.9	75.6	85.7
toPt-437866	70.1	61.8	57.1	73.6	67.3	45.2	53.5	67.4
pPt-473851	52.4	31.7	29.3	41.2	39.7	28.3	27.7	38.4
pPt-458887	50.1	28.4	25.0	36.7	35.7	22.9	23.5	32.9

All the markers are located on chromosome X and are inherited from the resistant parent 99-10/36

## Discussion

The *Rpi-rzc1 *gene from *S. ruiz-ceballosii*, that confers resistance to *P. infestans*, was mapped to potato chromosome X. The gene provides a high level of resistance in both potato foliage and tubers. The influence of field and testing conditions on results of leaflet and tuber slice tests was significant but weak (Table 3) which showed stable expression of the *Rpi-rzc1 *gene. Out of 130 *P. infestans *isolates, collected from various locations in Poland in the years 2007-2009, that were tested on *S. ruiz-ceballosii *plants, only 4 (3.1%) were virulent (Śliwka, unpublished), indicating the usefulness of the gene for potato breeding. Results of the field test for resistance to late blight under natural infection pressure of 13 individuals showed that gene *Rpi-rzc1 *confers resistance to *P. infestans *both in laboratory tests and in natural conditions.

The *Rpi-rzc1 *is a third gene for resistance to *P. infestans *that has been mapped to potato chromosome X. The first was an R gene originating from *S. berthaultii *and mapped 10.4 cM north from the marker TG403 [14]. The same gene, later named *R_Pi-ber_*, was mapped more precisely to a distance of 4.6 cM from the marker TG403 [15]. The *Rpi-rzc1 *gene could be in a similar location since, on the common CP map of dH Balbina × 99-10/36, it was 8.1 cM north from the marker TG403 (Additional file 2). When isolates of *P. infestans *compatible with *R_Pi-ber _*were used for inoculation, a smaller, but significant resistance effect was detected in the same map position as the R gene. This could be explained by the residual effect of the gene or/and a resistance QTL located in the same position [43]. The *R_Pi-ber _*gene proved to be effective in both foliage and tubers [44], similarly to *R1*, *R3b *and *Rpi-phu1 *[9,45,46] and also to *Rpi-rzc1 *(Figure 2, Table 4). In a breeding experiment on the effects of pyramiding of the R genes, mentioned in the introduction, *R_Pi-ber _*was used, together with the *R_Pi-mcd1 _*gene, and both genes showed an additive effect on resistance to late blight in a field test. A larger effect was provided by the *R_Pi-ber_*, which produced a three week delay in infection reaching 50% of the leaf area [17]. In the same species, *S. berthaultii*, two more R genes, *Rpi-ber1 *and *Rpi-ber2*, were identified and mapped to chromosome X [16]. Park *et al*. speculate that, on the basis of location and origin, *Rpi-ber1 *[16] may be identical to *R_Pi-ber _*[15]. *Rpi-ber2 *is most likely a different gene, although located in the similar position 12 cM north from the marker TG403 [16].

Apart from R genes, a number of QTL for resistance to *P. infestans *have been mapped to potato chromosome X [25,34,47-49]. These QTL originate from various *Solanum *species, including *S*. *sparsipilum*, which is a synonym of *S. ruiz-ceballosii *[25]. A QTL meta-analysis that allowed more precise comparisons of different genetic maps has identified two meta-QTL for late blight resistance on potato chromosome X. The *Rpi-ber1*/*R_Pi-ber _*is located between them and the *Rpi-ber2 *localization overlaps with a meta-QTL named MQTL_2_Late_blight [50]. In tomato, an incompletely dominant R gene allele, *Ph-2 *that originates from *S. pimpinellifolium *and confers resistance to *P. infestans*, has been mapped to chromosome X [51]. It's location in the close vicinity of the marker CT124 [51] is similar to the location of the *R_Pi-ber _*gene which is 4.6 cM away from the same marker [15]. In general, on the basis of mapping data, we cannot exclude the possibility that *Rpi-rzc1*, *Rpi-ber1*/*R_Pi-ber_*, *Rpi-ber2*, QTL for late blight resistance listed above and even the tomato *Ph-2 *gene are homologous and occupy the same locus on chromosome X.

An allele-specific molecular marker T1521 was located at a distance of 6.1 cM from the *Rpi-rzc1 *gene and this easy to score marker can be useful for marker-assisted selection (MAS) of resistant potatoes. DArT marker toPt-437866, located on the other side of the gene at a distance of 9.1 cM, can be transformed into a PCR marker and also used in MAS. The linkage of the *Rpi-rzc1 *gene to violet flower colour can be exploited for the selection of the resistant individuals only in certain breeding combinations. We called the colour observed in the mapping population "violet", following the botanical description of the species [18], although it may correspond to the blue or purple flower colour described in other potato germplasm [30,31]. The violet flower colour segregated in a clear 1:1 ratio in the mapping population dH Balbina × 99-10/36 indicating that this trait was controlled by a single dominant allele, heterozygous in the *S. ruiz-ceballosii *99-10/36 parent. The chromosomal position of its locus suggested that most likely it was the previously mapped *F *locus and showed it to be involved in the flower colour expression in combination with the locus *P *or *D *[30,31]. The presence of at least a single dominant *F *allele and *P *or *D *allele is required to turn potato flowers from white to blue or red, respectively [30]. The presence of the both *D *and *P *alleles together with the *F *allele resulted in the purple flower colour [31]. We can only deduce the genotypes of the parents in the *F *locus as dH Balbina: *ff *and 99-10/36: *Ff*, on the basis of the segregation and chromosomal position of the flower colour locus. Within the progeny, three different shades/intensities of the violet colour were observed indicating the segregation of various numbers of *D *and/or *P *alleles, also possibly inherited from dH Balbina, in which the *ff *genotype would mask their presence. Flower colour could serve as a phenotypic marker of late blight resistance in other progenies with the *Rpi-rzc1 *gene only when the susceptible parent would have an ability to synthesize anthocyanin pigments (*D *and/or *P *alleles) but not expressed in flowers (genotype *ff*).

The DArT markers applied in this study are a cost-effective, quick and efficient tool for genotyping and they are being used more and more frequently for mapping studies in diverse plant species, like cotton [52], pearl millet [53], pigeon pea [54], eucalyptus [40], rye [55] and many others. The first genetic map of a potato relative constructed with the use of DArT markers was made for *S. bulbocastanum *(1 EBN). Twelve linkage groups consisting of 439 markers were obtained with a total map length of 403 cM. However, so far those linkage groups have not been assigned to potato chromosomes [56]. A DArT linkage map was also made for *S. michoacanum *(1 EBN). It consisted of 846 DArT markers and 48 sequence-specific PCR markers with known locations that allowed the identification of all particular potato chromosomes [36]. Recently, a linkage map of 2 EBN *Solanum *species containing, among others, DArT markers, has been published under a name: *Solanum phureja *diploid map 2010 [42]. It was made within the Potato Genome Sequencing Project in order to anchor and orientate physical contigs along the chromosomes [57]. A comparison of the dH Balbina × *S. ruiz-ceballosii *99-10/36 map with the *Solanum phureja *diploid map 2010 [42] and the *S. michoacanum *map [36] is summarized in Table 4 and in the Additional file 2. Out of 1,603 DArT markers located on our map, only 361 (22.5%) were also present on the *Solanum phureja *diploid map 2010 [42], most of them (332 i.e. 92%) in similar positions on both maps (Table 4). Even fewer 137 (8.5%) DArT markers were common to both *S. ruiz-ceballosii *and *S. michoacanum *[36] linkage maps, but even so the majority of them located in concordant positions (122, i.e.89%). These results suggest that even though different DArT markers segregate in different mapping populations, there is clearly synteny within the *Solanum *genus as very few markers were mapped in discordant positions on the three maps. The numbers of common and similarly located markers between these three maps confirmed that indeed potatoes from the *S. tuberosum *× *S. ruiz-ceballosii *cross are more closely related to *S. phureja *than to the 1 EBN species *S. michoacanum*.

## Conclusions

The *Rpi-rzc1 *gene from *S. ruiz-ceballosii*, that confers resistance to *P. infestans*, was mapped to potato chromosome X. The gene provides a high level of resistance in both foliage and tubers. The gene has already been introduced into the cultivated potato gene pool via the interspecific cross described here. Knowledge of the chromosomal localization of the *Rpi-rzc1 *gene can be useful for designing gene pyramids. Molecular markers identified in our study can support marker-assisted selection of individuals possessing the gene, as can also the phenotypic marker violet flower colour. We constructed a genetic linkage map for dH Balbina (*S. tuberosum*) × 99-10/36 (*S. ruiz-ceballosii*) using 1,603 DArT markers and 48 reference sequence-specific PCR markers of known chromosomal localization. This is the first DArT map for both species and one of the first within the *Solanum *genus. Out of these 1,603 DArT markers, 1,149 were mapped for the first time in a *Solanum *plant, providing a useful resource for new DArT mapping studies that will enable map comparisons, unambiguous chromosome identification and orientation, as well as validation of marker positions.

## Authors' contributions

JŚ participated in the resistance testing, reference marker search, carried out statistical and linkage analyses and drafted the manuscript. HJ selected the parental clones and obtained the mapping population, assessed the flower colour and participated in the resistance testing. MC, IT and AH-S took part in search and scoring of the reference markers. AK was responsible for the DArT analyses. EZ-G participated in the design of the study and its coordination. All authors read and approved the final manuscript.

## Authors' information

Professor EZ-G is a Head of Młochów Research Centre within Plant Breeding and Acclimatization Institute -National Research Institute. Doctor JŚ leads a Phytopathology Laboratory and Doctor HJ leads Genetics Laboratory, while MC, AH-S and IT are PhD students in the same institution. Doctor AK is a Director of Diversity Arrays Technology Pty Ltd.

## Supplementary Material

Additional file 1 **Sequence-specific markers used for the construction of genetic maps of the *S*. *ruiz-ceballosii *clone 99-10/36 and potato dihaploid of cv. Balbina**. [7,15,16,34,42,58-61]. Click here for file

Additional file 2 **Genetic map of the dH Balbina × *S. ruiz-ceballosii *99-10/36 population constructed using JoinMap^® ^4 software**. Click here for file
