# Supplementary material for: Late blight resistance gene from Solanum ruiz-ceballosii is located on potato chromosome X and linked to violet flower colour
Source: BMC Genet. 2012 Feb 27;13:11. doi: 10.1186/1471-2156-13-11 (PMC3347998; doi:10.1186/1471-2156-13-11)
Supplement: Additional file 1 — Sequence-specific markers used for the construction of genetic maps of the S. ruiz-ceballosii clone 99-10/36 and potato dihaploid of cv. Balbina. [7,15,16,34,42,58-61]. [file 1471-2156-13-11-S1.DOC]

Additional file 1 Sequence-specific markers used for the construction of genetic maps of the *S. ruiz-ceballosii* clone 99-10/36 and potato dihaploid of cv. Balbina

| Chr. | Name of the marker | Primer sequences | Ta | Size  (bp) | Restriction enzyme | Parent | Source |
| --- | --- | --- | --- | --- | --- | --- | --- |
| I | BA114i24t7 | GAAGTGCTCACCAGAAACCAAG | 55 | 500/550/ | - | 99-10/36 | [58] |
|  |  | TTCTGATTTGCCCTCGATTTGTC |  | 575/625 |  | dH Balbina |  |
|  | C2_At5g51970 | ACGCGAGTTTTGACTGTGCTGG | 58 | 1000 | *Xap*I | dH Balbina | [42] |
|  |  | TCTTCTTGAGAGAATCCAAACCTGTG |  |  |  |  |  |
|  | C2_At4g38810 | AGGTGATGGACGGTTCAGATATAATG | 55 | 1500 | *Fsp*BI | 99-10/36 | [42] |
|  |  | AACTTCTTTAAACTCAGTCTTGCTTAC |  |  |  |  |  |
|  | C2_At5g64350 | AGATCGGCCAAGGCAAAGTTATC | 55 | 850 | *Hin*dIII | 99-10/36 | [42] |
|  |  | TGCATGCCCAGTACTCCTTCATCC |  |  |  |  |  |
| II | C2_At2g18030 | TTGGGCGACCACGCTGAATC | 55 | 1300 | *Msp*I | dH Balbina | [42] |
|  |  | TTACCCACATCAGGACCTTGCC |  |  |  |  |  |
|  | C2_At5g66090 | ATCTCTCTGAGGGTTCAAGACAGG | 55 | 500/ | - | dH Balbina | [42] |
|  |  | TATATCAGCTCCATACTTCTTTGC |  | 1100 |  |  |  |
|  | C2_A4g30930 | ATCATACCTTCTCTCTCCAAACCC | 55 | 750 | *Bsu*RI | 99-10/36 | [42] |
|  |  | TCGCCATTGCTCACTTTAAACTG |  |  |  |  |  |
|  | C2_At4g05090 | AGTATTATAGCGAGCTCGAAGCTGC | 55 | 1600 | *Msp*I | 99-10/36 | [42] |
|  |  | TCCTTTCCCCCTCTGTCAATTGC |  |  |  |  |  |
| III | C2_At3g10220 | TGGCTTCTCAGTTACAGATTCAAGG | 55 | 1100 | *Alu*I | dH Balbina | [42] |
|  |  | AACCTCCGGAGGTCACTGACG |  |  |  |  |  |
|  | C2_At3g03100 | TGGTGCAACACTTGTTGGTGTGG | 55 | 900 | *Tru*1I | 99-10/36 | [42] |
|  |  | TGGAGCCAGCCATGCCATTC |  |  |  |  |  |
|  | C2_At5g62390 | TGCTACTAACTGTTGATGCCATTGAG | 55 | 1000 | *Xap*I | dH Balbina | [42] |
|  |  | TTGGGGGTCGATAACATCAAGC |  |  |  | 99-10/36 |  |
| IV | C2_At3g17040 | TGGGGTTGGATGGAGTGGAAAG | 55 | 500 | *Eco*RI | dH Balbina | [42] |
|  |  | AGTAGAGGTTACGAATTTCCTCTGC |  |  |  |  |  |
|  | TG370 | TCGAAGCTCTGTTTCTGCTC | 55 | 300 | *Taq*I | dH Balbina | [46] |
|  |  | CCCATGTTATGCCATTCAGC |  |  |  |  |  |
|  | C2_At1g05055^a^ | CTTAACAGATCTTGGTGGCAGTCC | 55 | 800 | *Bsu*RI | 99-10/36 | [42] |
|  |  | TGTATCCACCGCCAACTTTAGC |  |  |  |  |  |
| V | GP78 | GCATATCACCAACGATTGCT | 55 | 1000/ | - | dH Balbina | [34] |
|  |  | CAGGAGGCTCAAAACTTTCA |  | 1200 |  |  |  |
|  | C2_At1g14790 | TGGAGAGAACACTAATATTCTCAAGG | 55 | 850/ | - | 99-10/36 | [42] |
|  |  | ATGTCTTGATCCCAGCAAACAAAG |  | 1000 |  |  |  |
|  | U227536 | CTGAATGCTCTGTGCTCGAC | 55 | 1300 | *Bsu*RI | 99-10/36 | [42] |
|  |  | TCCTCATCTCTGGGGTTCAC |  |  |  |  |  |
| VI | 24L | GAGATTCTCAAAGGTGTCTTCC | 55 | 450 | *Tru*1I | dH Balbina | [59] |
|  |  | AACCTGTGCTTTCCCATTCG |  |  |  |  |  |
|  | C2_At2g28690 | ATGGAAGAATTTGATTCTTTATGGAG | 55 | 1500 | *Hin*dIII | dH Balbina | [42] |
|  |  | TGATCTTTTGCTTCATCTCTTTCTTG |  |  | *Rsa*I | 99-10/36 |  |
| VII | C2_At1g53670 | AAGGGTACAGAACGGGCATTCAC | 55 | 1000 | *Eco*RI | 99-10/36 | [42] |
|  |  | TGTTCCAGGGGTCTTACTGTTCCAG |  |  |  | dH Balbina |  |
|  | C2_At3g15290 | TCTGCTATTTTGGCTTCTAATACAAG | 55 | 900 | *Tru*1I | 99-10/36 | [42] |
|  |  | ACAATATGTGTCTTCTGATGTATCTGC |  |  |  |  |  |
|  | C2_At3g15430 | TCGTTTGAGGTCAACTTTAATGGAGG | 55 | 1000 | *Rsa*I | dH Balbina | [42] |
|  |  | AGTTGTTTCAGGCCCATGACCAAG |  |  |  |  |  |
|  | C2_At2g42810 | TTGCCTTCAAGTGCATGTGTCC | 55 | 800 | *Bsu*RI | dH Balbina | [42] |
|  |  | AGAGAGCTTCACGCCATCAACAC |  |  |  |  |  |
| VIII | GP92 | AAGGGTGTTGAAGAAGC | 55 | 300 | - | dH Balbina | Marczewski |
|  |  | CAGGGAAGGAAAAGGAAC |  |  |  |  | unpublished |
|  | GP189 | AGTTGAGGAGCTGTTTGTGA | 55 | 2500 | *Eco*RI | dH Balbina | [60] |
|  |  | AGGCTTTAGTATTTCTGTGTATTT |  |  |  |  |  |
|  | C2_At4g31115 | ACTTCTAAATTTTGAAGTTGCCCCTG | 55 | 2500/2800 | - | 99-10/36 | [42] |
|  |  | AGAAGTAGGCAGCATGGTGAATGG |  |  |  |  |  |
|  | C2_At4g32770 | TCTTCTATGAATTTGTTCCATGGAATG | 55 | 650 | *Taq*I | 99-10/36 | [42] |
|  |  | ACCATGGTCCTCCCCCAACTTC |  |  |  |  |  |
| IX | C2_At2g47590 | ACGAGCGTCGATTGTTTGGTTCC | 55 | 550 | *Alu*I | dH Balbina | [42] |
|  |  | ACTAGGATTGAGCCCCAAATCAACC |  |  |  |  |  |
|  | GP254 | AGTGCACCAAGGGTGTGAC | 55 | 1800 | *Tru*1I | dH Balbina | [7] |
|  |  | AAGTGCATGCCTGTAATGGC |  |  |  |  |  |
|  | Svnt13M5.17 | CTGAGGTGCAGCCAATAAC | 55 | 500 | - | dH Balbina | [7] |
|  |  | CCAGTGAGAAACAGCTTCTC |  |  |  |  |  |
|  | GP129 | GTGGTAGCAAAGTATTCATC | 55 | 1000 | *Fsp*BI | 99-10/36 | [58] |
|  |  | CGTTATCTGGACTCCTTTAG |  |  |  |  |  |
| X | TG403 | AGCTGTCAGAAAGATTGGGAG | 55 | 2800 | *Taq*I | dH Balbina | [15] |
|  |  | GCATTTGCATCAAGTGGTTC |  |  |  |  |  |
|  | C2_At2g37510 | TGGCAATTATGGCATTATTTCGTCG | 55 | 2000 | *Hha*I | dH Balbina | [42] |
|  |  | AGGTTTTCATCACCAGTGTATCTTGAAAG |  |  |  |  |  |
|  | CT240 | CCAAAGCCCAGGCTGTCAAG | 55 | 900 | *Hin*fI | 99-10/36 | [14] |
|  |  | AGTCGGGTGTCACAATAA |  |  |  |  |  |
|  | T1521 | CAAGTATGGCAGGAACAAGTAA | 55 | 850/950/ | - | 99-10/36 | Marczewski |
|  |  | ATAGACGACGAATTTCCAGCATA |  | 1800/2000 |  |  | unpublished |
| XI | T0302 | TGGCTCATCCTGAAGCTGATAGCGC | 55 | 600 | *Taq*I | 99-10/36 | [42] |
|  |  | AGTGTACATCCTTGCCATTGACT |  |  |  |  |  |
|  | T0302 | TGGCTCATCCTGAAGCTGATAGCGC | 55 | 500 | *Taq*I | 99-10/36 | [42] |
|  |  | AGTGTACATCCTTGCCATTGACT |  |  |  |  |  |
|  | TG105 | TCACATGAGCTGGGAGAAAT | 55 | 600 | *Tru*1I | 99-10/36 | [61] |
|  |  | AAAGGCCTGTTGCTGAGAG |  |  |  |  |  |
|  | GP185 | CTGGTAATAGTAGTAATGATTCTTCGTC | 55 | 400 | *Taq*I | dH Balbina | [61] |
|  |  | TTGTTCAATGGAGCACTTGC |  |  |  |  |  |
|  | T0302 | TGGCTCATCCTGAAGCTGATAGCGC | 55 | 550 | *Fsp*BI | dH Balbina | [42] |
| XII | GP200 | AAGCATCTGCTACCTGCAATAGAA | 55 | 1800 | *Xap*I | dH Balbina | Marczewski |
|  |  | GTGTGGCAAAACTCTGAAAATG |  |  |  |  | unpublished |
|  | GP268 | ATGCTTGAACCCGAAGTAACG | 55 | 500 | *Hha*I | dH Balbina | Marczewski |
|  |  | TTGAAATACCGTATGTCTTTGTGC |  |  |  |  | unpublished |
|  | GP197 | AATCAGAAAAGAAGTTGTGTTGG | 55 | 2300 | - | 99-10/36 | Marczewski |
|  |  | ATGTAGTAAGGGATCGGTTCGTT |  |  |  |  | unpublished |
|  | GP229 | AAATATTTGGGTTCCTCTTG | 55 | 700 | *Rsa*I | 99-10/36 | Marczewski |
|  |  | AAAAATTATCTTCACAGCAGTAG |  |  |  |  | unpublished |
|  | TG618 | CATGCCTGCAGAACACATACA | 55 | 450 | *Tas*I | 99-10/36 | Marczewski |
|  |  | TGTCACCTACTTGTTTTGGTTTGT |  |  |  |  | unpublished |

^a^ a marker that in population dH Balbina x 99-10/36 was mapped to chromosome VI.
