# Supplementary material for: Late blight resistance gene from Solanum ruiz-ceballosii is located on potato chromosome X and linked to violet flower colour
Source: BMC Genet. 2012 Feb 27;13:11. doi: 10.1186/1471-2156-13-11 (PMC3347998; doi:10.1186/1471-2156-13-11)
Supplement: Additional file 2 — Genetic map of the dH Balbina × S. ruiz-ceballosii 99-10/36 population constructed using JoinMap® 4 software. [file 1471-2156-13-11-S2.PPT]

## Slide 1
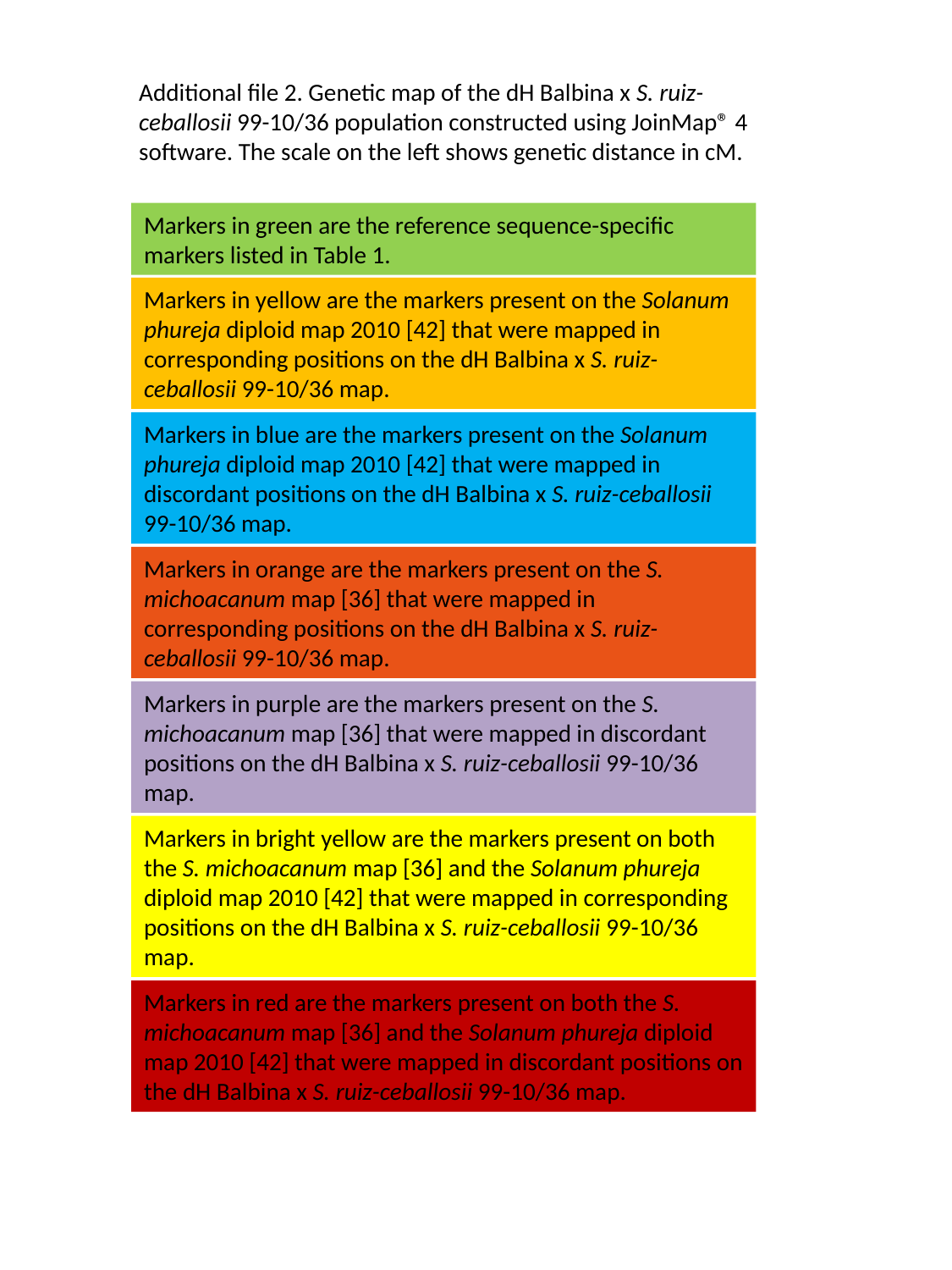

Additional file 2. Genetic map of the dH Balbina x S. ruiz-ceballosii 99-10/36 population constructed using JoinMap® 4 software. The scale on the left shows genetic distance in cM.
Markers in green are the reference sequence-specific markers listed in Table 1.
Markers in yellow are the markers present on the Solanum phureja diploid map 2010 [42] that were mapped in corresponding positions on the dH Balbina x S. ruiz-ceballosii 99-10/36 map.
Markers in blue are the markers present on the Solanum phureja diploid map 2010 [42] that were mapped in discordant positions on the dH Balbina x S. ruiz-ceballosii 99-10/36 map.
Markers in orange are the markers present on the S. michoacanum map [36] that were mapped in corresponding positions on the dH Balbina x S. ruiz-ceballosii 99-10/36 map.
Markers in purple are the markers present on the S. michoacanum map [36] that were mapped in discordant positions on the dH Balbina x S. ruiz-ceballosii 99-10/36 map.
Markers in bright yellow are the markers present on both the S. michoacanum map [36] and the Solanum phureja diploid map 2010 [42] that were mapped in corresponding positions on the dH Balbina x S. ruiz-ceballosii 99-10/36 map.
Markers in red are the markers present on both the S. michoacanum map [36] and the Solanum phureja diploid map 2010 [42] that were mapped in discordant positions on the dH Balbina x S. ruiz-ceballosii 99-10/36 map.

## Slide 2
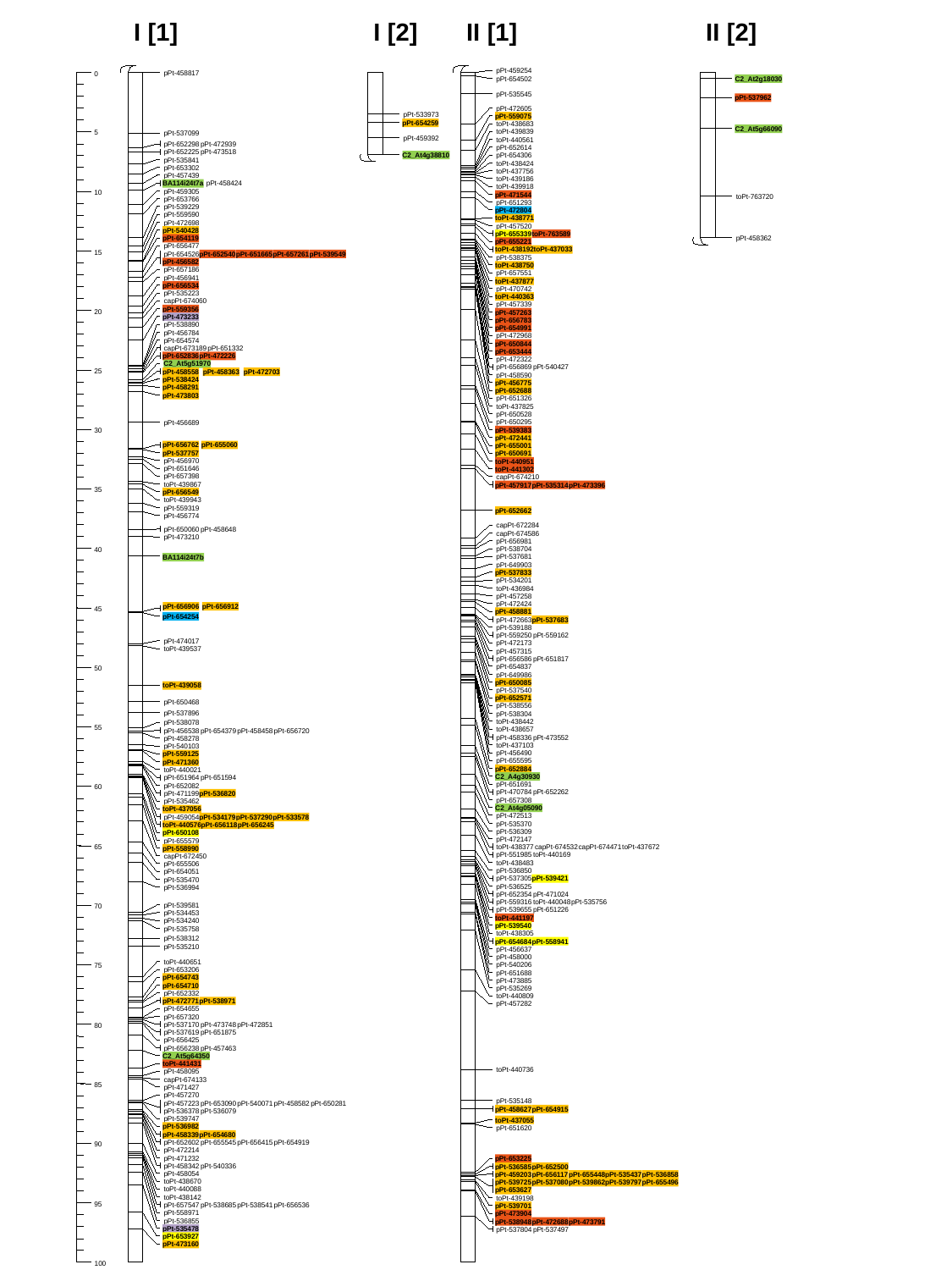

I [1]
pPt-458817
pPt-537099
pPt-652298
pPt-472939
pPt-652225
pPt-473518
pPt-535841
pPt-653302
pPt-457439
pPt-458424
BA114i24t7a
pPt-459305
pPt-653766
pPt-539229
pPt-559590
pPt-472698
pPt-540428
pPt-654119
pPt-656477
pPt-654526
pPt-652540
pPt-651665
pPt-657261
pPt-539549
pPt-456582
pPt-657186
pPt-456941
pPt-656534
pPt-535223
capPt-674060
pPt-559356
pPt-473233
pPt-538890
pPt-456784
pPt-654574
capPt-673189
pPt-651332
pPt-652836
pPt-472226
C2_At5g51970
pPt-458558
pPt-458363
pPt-472703
pPt-538424
pPt-458291
pPt-473803
pPt-456689
pPt-656762
pPt-655060
pPt-537757
pPt-456970
pPt-651646
pPt-657398
toPt-439867
pPt-656549
toPt-439943
pPt-559319
pPt-456774
pPt-650060
pPt-458648
pPt-473210
pPt-656906
pPt-656912
pPt-654254
pPt-474017
toPt-439537
toPt-439058
pPt-650468
pPt-537896
pPt-538078
pPt-456538
pPt-654379
pPt-458458
pPt-656720
pPt-458278
pPt-540103
pPt-559125
pPt-471360
toPt-440021
pPt-651964
pPt-651594
pPt-652082
pPt-471199
pPt-536820
pPt-535462
toPt-437056
pPt-459054
pPt-534179
pPt-537290
pPt-533578
toPt-440576
pPt-656118
pPt-656245
pPt-650108
pPt-655579
pPt-558990
capPt-672450
pPt-655506
pPt-654051
pPt-535470
pPt-536994
pPt-539581
pPt-534453
pPt-534240
pPt-535758
pPt-538312
pPt-535210
toPt-440651
pPt-653206
pPt-654743
pPt-654710
pPt-652332
pPt-472771
pPt-538971
pPt-654655
pPt-657320
pPt-537170
pPt-473748
pPt-472851
pPt-537619
pPt-651875
pPt-656425
pPt-656238
pPt-457463
C2_At5g64350
toPt-441431
pPt-458095
capPt-674133
pPt-471427
pPt-457270
pPt-457223
pPt-653090
pPt-540071
pPt-458582
pPt-650281
pPt-536378
pPt-536079
pPt-539747
pPt-536982
pPt-458339
pPt-654680
pPt-652602
pPt-655545
pPt-656415
pPt-654919
pPt-472214
pPt-471232
pPt-458342
pPt-540336
pPt-458054
toPt-438670
toPt-440088
toPt-438142
pPt-657547
pPt-538685
pPt-538541
pPt-656536
pPt-558971
pPt-536855
pPt-535478
pPt-653927
pPt-473160
I [2]
pPt-533973
pPt-654259
pPt-459392
C2_At4g38810
II [1]
pPt-459254
pPt-654502
pPt-535545
pPt-472605
pPt-559075
toPt-438683
toPt-439839
toPt-440561
pPt-652614
pPt-654306
toPt-438424
toPt-437756
toPt-439186
toPt-439918
pPt-471544
pPt-651293
pPt-472804
toPt-438771
pPt-457520
pPt-655339
toPt-763589
pPt-655221
toPt-438192
toPt-437033
pPt-538375
toPt-438750
pPt-657551
toPt-437877
pPt-470742
toPt-440363
pPt-457339
pPt-457263
pPt-656783
pPt-654991
pPt-472968
pPt-650844
pPt-653444
pPt-472322
pPt-656869
pPt-540427
pPt-458590
pPt-456775
pPt-652688
pPt-651326
toPt-437825
pPt-650528
pPt-650295
pPt-539383
pPt-472441
pPt-655001
pPt-650691
toPt-440951
toPt-441302
capPt-674210
pPt-457917
pPt-535314
pPt-473396
pPt-652662
capPt-672284
capPt-674586
pPt-656981
pPt-538704
pPt-537681
pPt-649903
pPt-537833
pPt-534201
toPt-436984
pPt-457258
pPt-472424
pPt-458881
pPt-472663
pPt-537683
pPt-539188
pPt-559250
pPt-559162
pPt-472173
pPt-457315
pPt-656586
pPt-651817
pPt-654837
pPt-649986
pPt-650085
pPt-537540
pPt-652571
pPt-538556
pPt-538304
toPt-438442
toPt-438657
pPt-458336
pPt-473552
toPt-437103
pPt-456490
pPt-655595
pPt-652884
C2_A4g30930
pPt-651691
pPt-470784
pPt-652262
pPt-657308
C2_At4g05090
pPt-472513
pPt-535370
pPt-536309
pPt-472147
toPt-438377
capPt-674532
capPt-674471
toPt-437672
pPt-551985
toPt-440169
toPt-438483
pPt-536850
pPt-537305
pPt-539421
pPt-536525
pPt-652354
pPt-471024
pPt-559316
toPt-440048
pPt-535756
pPt-539655
pPt-651226
toPt-441197
pPt-539540
toPt-438305
pPt-654684
pPt-558941
pPt-456637
pPt-458000
pPt-540206
pPt-651688
pPt-473885
pPt-535269
toPt-440809
pPt-457282
toPt-440736
pPt-535148
pPt-458627
pPt-654915
toPt-437055
pPt-651620
pPt-653225
pPt-536585
pPt-652500
pPt-459203
pPt-656117
pPt-655448
pPt-535437
pPt-536858
pPt-539725
pPt-537080
pPt-539862
pPt-539797
pPt-655496
pPt-653627
toPt-439198
pPt-539701
pPt-473904
pPt-538948
pPt-472688
pPt-473791
pPt-537804
pPt-537497
II [2]
C2_At2g18030
pPt-537962
C2_At5g66090
toPt-763720
pPt-458362
0
5
10
15
20
25
30
35
40
45
50
55
60
65
70
75
80
85
90
95
100
BA114i24t7b

## Slide 3
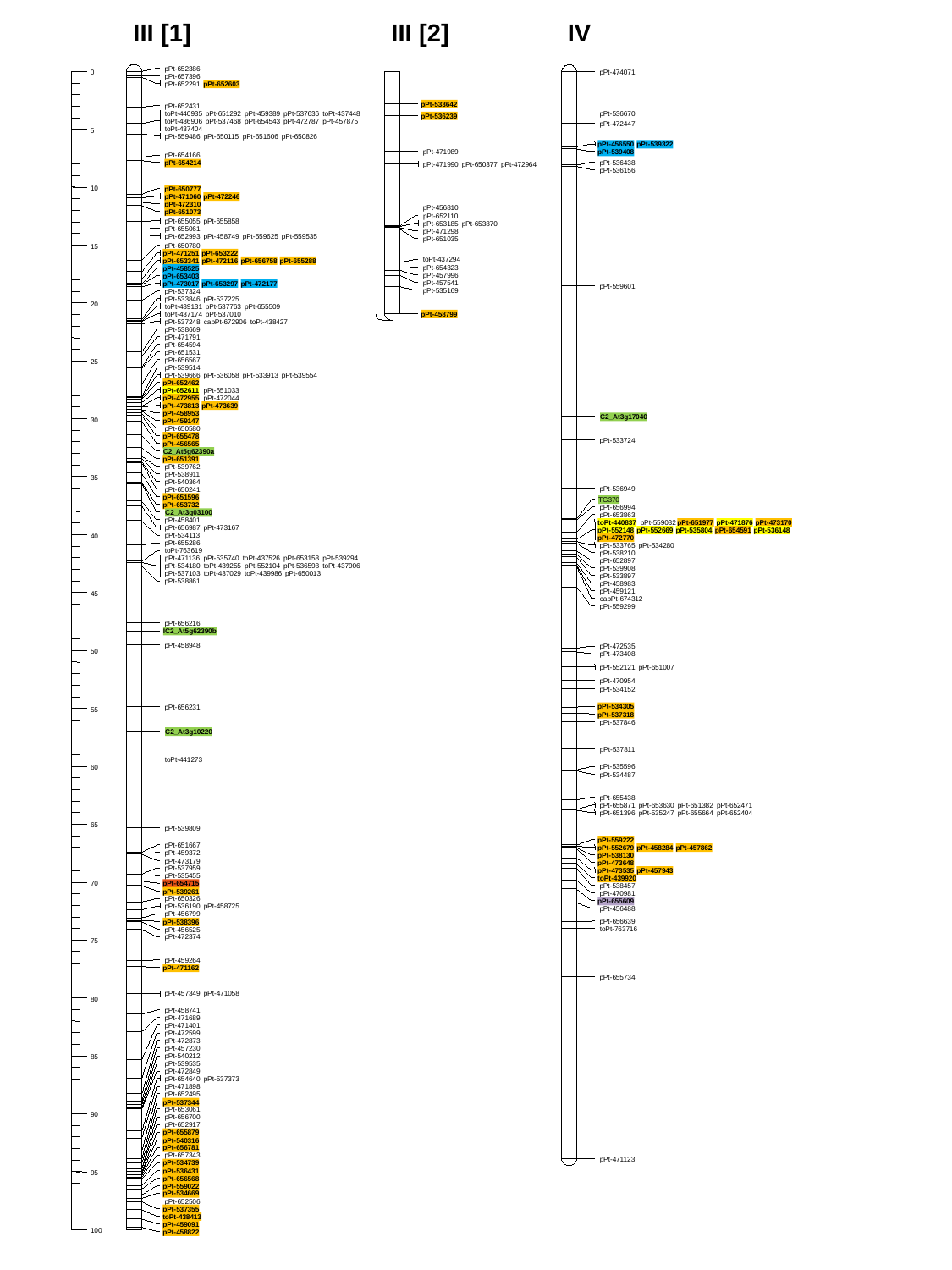

III [1]
pPt-652386
pPt-657396
pPt-652291
pPt-652603
pPt-652431
toPt-440935
pPt-651292
pPt-459389
pPt-537636
toPt-437448
toPt-436906
pPt-537468
pPt-654543
pPt-472787
pPt-457875
toPt-437404
pPt-559486
pPt-650115
pPt-651606
pPt-650826
pPt-654166
pPt-654214
pPt-650777
pPt-471060
pPt-472246
pPt-472310
pPt-651073
pPt-655055
pPt-655858
pPt-655061
pPt-652993
pPt-458749
pPt-559625
pPt-559535
pPt-650780
pPt-471251
pPt-653222
pPt-653341
pPt-472116
pPt-656758
pPt-655288
pPt-458525
pPt-653403
pPt-473017
pPt-653297
pPt-472177
pPt-537324
pPt-533846
pPt-537225
toPt-439131
pPt-537763
pPt-655509
toPt-437174
pPt-537010
pPt-537248
capPt-672906
toPt-438427
pPt-538669
pPt-471791
pPt-654594
pPt-651531
pPt-656567
pPt-539514
pPt-539666
pPt-536058
pPt-533913
pPt-539554
pPt-652462
pPt-652611
pPt-651033
pPt-472955
pPt-472044
pPt-473813
pPt-473639
pPt-458953
pPt-459147
pPt-650580
pPt-655478
pPt-456565
C2_At5g62390a
pPt-651391
pPt-539762
pPt-538911
pPt-540364
pPt-650241
pPt-651596
pPt-653732
C2_At3g03100
pPt-458401
pPt-656987
pPt-473167
pPt-534113
pPt-655286
toPt-763619
pPt-471136
pPt-535740
toPt-437526
pPt-653158
pPt-539294
pPt-534180
toPt-439255
pPt-552104
pPt-536598
toPt-437906
pPt-537103
toPt-437029
toPt-439986
pPt-650013
pPt-538861
pPt-656216
IC2_At5g62390b
pPt-458948
pPt-656231
C2_At3g10220
toPt-441273
pPt-539809
pPt-651667
pPt-459372
pPt-473179
pPt-537959
pPt-535455
pPt-654715
pPt-539261
pPt-650326
pPt-536190
pPt-458725
pPt-456799
pPt-538396
pPt-456525
pPt-472374
pPt-459264
pPt-471162
pPt-457349
pPt-471058
pPt-458741
pPt-471689
pPt-471401
pPt-472599
pPt-472873
pPt-457230
pPt-540212
pPt-539535
pPt-472849
pPt-654640
pPt-537373
pPt-471898
pPt-652495
pPt-537344
pPt-653061
pPt-656700
pPt-652917
pPt-655879
pPt-540316
pPt-656781
pPt-657343
pPt-534739
pPt-536431
pPt-656568
pPt-559022
pPt-534669
pPt-652506
pPt-537355
toPt-438413
pPt-459091
pPt-458822
III [2]
pPt-533642
pPt-536239
pPt-471989
pPt-471990
pPt-650377
pPt-472964
pPt-456810
pPt-652110
pPt-653185
pPt-653870
pPt-471298
pPt-651035
toPt-437294
pPt-654323
pPt-457996
pPt-457541
pPt-535169
pPt-458799
IV
pPt-474071
pPt-536670
pPt-472447
pPt-456550
pPt-539322
pPt-539408
pPt-536438
pPt-536156
pPt-559601
C2_At3g17040
pPt-533724
pPt-536949
TG370
pPt-656994
pPt-653863
toPt-440837
pPt-559032
pPt-651977
pPt-471876
pPt-473170
pPt-552148
pPt-552669
pPt-535804
pPt-654591
pPt-536148
pPt-472770
pPt-533765
pPt-534280
pPt-538210
pPt-652897
pPt-539908
pPt-533897
pPt-458983
pPt-459121
capPt-674312
pPt-559299
pPt-472535
pPt-473408
pPt-552121
pPt-651007
pPt-470954
pPt-534152
pPt-534305
pPt-537318
pPt-537846
pPt-537811
pPt-535596
pPt-534487
pPt-655438
pPt-655871
pPt-653630
pPt-651382
pPt-652471
pPt-651396
pPt-535247
pPt-655664
pPt-652404
pPt-559222
pPt-552679
pPt-458284
pPt-457862
pPt-538130
pPt-473648
pPt-473535
pPt-457943
toPt-439920
pPt-538457
pPt-470981
pPt-655609
pPt-456488
pPt-656639
toPt-763716
pPt-655734
pPt-471123
0
5
10
15
20
25
30
35
40
45
50
55
60
65
70
75
80
85
90
95
100

## Slide 4
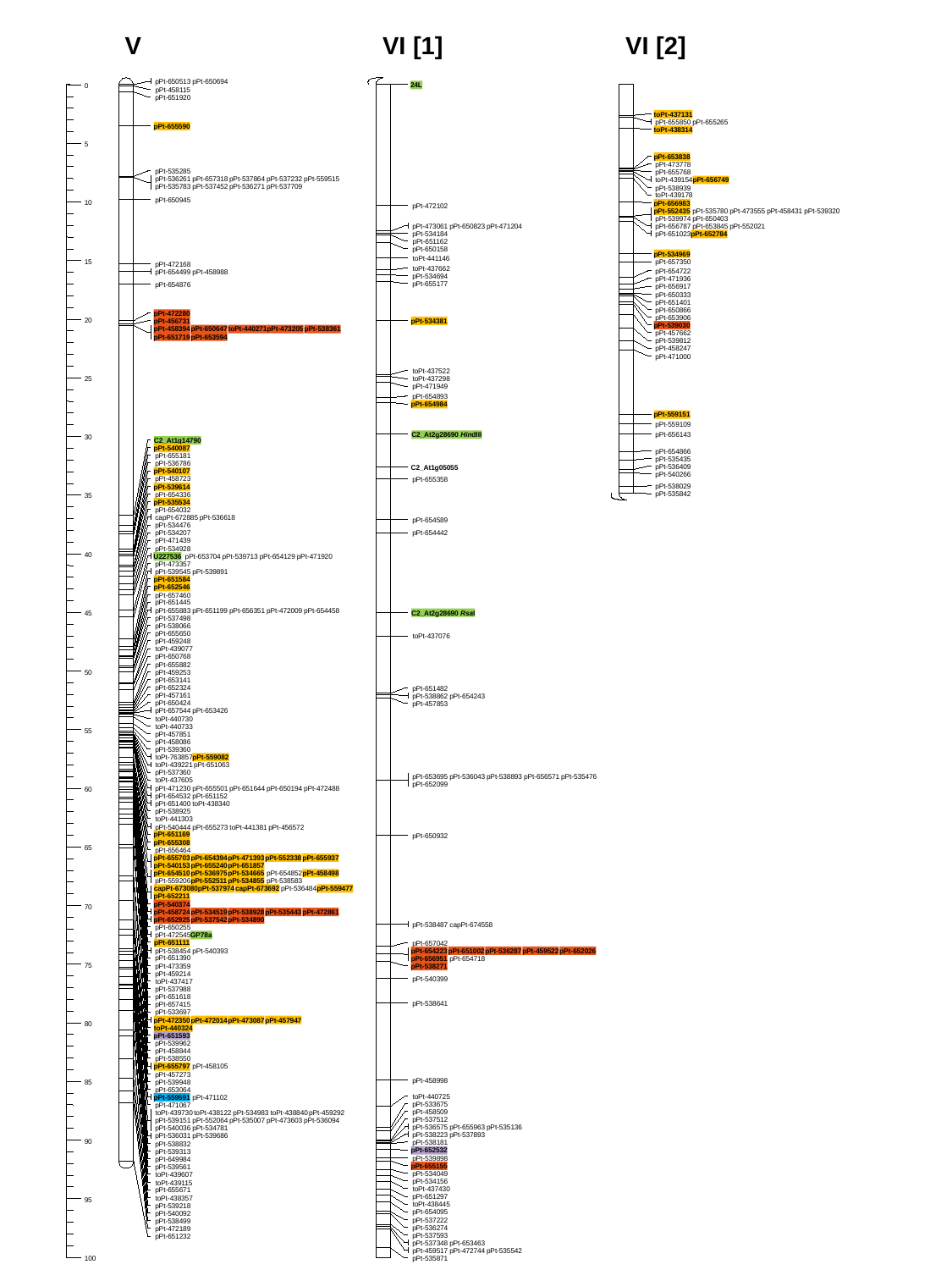

V
pPt-650513
pPt-650694
pPt-458115
pPt-651920
pPt-655590
pPt-535285
pPt-536261
pPt-657318
pPt-537864
pPt-537232
pPt-559515
pPt-535783
pPt-537452
pPt-536271
pPt-537709
pPt-650945
pPt-472168
pPt-654499
pPt-458988
pPt-654876
pPt-472280
pPt-456731
pPt-458394
pPt-650647
toPt-440271
pPt-473205
pPt-538361
pPt-651719
pPt-653594
C2_At1g14790
pPt-540087
pPt-655181
pPt-536786
pPt-540107
pPt-458723
pPt-539614
pPt-654336
pPt-535534
pPt-654032
capPt-672885
pPt-536618
pPt-534476
pPt-534207
pPt-471439
pPt-534928
U227536
pPt-653704
pPt-539713
pPt-654129
pPt-471920
pPt-473357
pPt-539545
pPt-539891
pPt-651584
pPt-652546
pPt-657460
pPt-651445
pPt-655883
pPt-651199
pPt-656351
pPt-472009
pPt-654458
pPt-537498
pPt-538066
pPt-655650
pPt-459248
toPt-439077
pPt-650768
pPt-655882
pPt-459253
pPt-653141
pPt-652324
pPt-457161
pPt-650424
pPt-657544
pPt-653426
toPt-440730
toPt-440733
pPt-457851
pPt-458086
pPt-539360
toPt-763857
pPt-559082
toPt-439221
pPt-651063
pPt-537360
toPt-437605
pPt-471230
pPt-655501
pPt-651644
pPt-650194
pPt-472488
pPt-654532
pPt-651152
pPt-651400
toPt-438340
pPt-538925
toPt-441303
pPt-540444
pPt-655273
toPt-441381
pPt-456572
pPt-651169
pPt-655308
pPt-656464
pPt-655703
pPt-654394
pPt-471393
pPt-552338
pPt-655937
pPt-540153
pPt-655240
pPt-651857
pPt-654510
pPt-536975
pPt-534665
pPt-654852
pPt-458498
pPt-559206
pPt-552511
pPt-534855
pPt-538583
capPt-673080
pPt-537974
capPt-673692
pPt-536484
pPt-559477
pPt-652211
pPt-540374
pPt-458724
pPt-534519
pPt-538928
pPt-535443
pPt-472861
pPt-652925
pPt-537542
pPt-534890
pPt-650255
pPt-472545
GP78a
pPt-651111
pPt-538454
pPt-540393
pPt-651390
pPt-473359
pPt-459214
toPt-437417
pPt-537988
pPt-651618
pPt-657415
pPt-533697
pPt-472350
pPt-472014
pPt-473087
pPt-457947
toPt-440324
pPt-651593
pPt-539962
pPt-458844
pPt-538550
pPt-655797
pPt-458105
pPt-457273
pPt-539948
pPt-653064
pPt-559591
pPt-471102
pPt-471067
toPt-439730
toPt-438122
pPt-534983
toPt-438840
pPt-459292
pPt-539151
pPt-552064
pPt-535007
pPt-473603
pPt-536094
pPt-540036
pPt-534781
pPt-536031
pPt-539686
pPt-538832
pPt-539313
pPt-649984
pPt-539561
toPt-439607
toPt-439115
pPt-655671
toPt-438357
pPt-539218
pPt-540092
pPt-538499
pPt-472189
pPt-651232
VI [1]
24L
pPt-472102
pPt-473061
pPt-650823
pPt-471204
pPt-534184
pPt-651162
pPt-650158
toPt-441146
toPt-437662
pPt-534694
pPt-655177
pPt-534381
toPt-437522
toPt-437298
pPt-471949
pPt-654893
pPt-654984
C2_At2g28690 HindIII
C2_At1g05055
pPt-655358
pPt-654589
pPt-654442
C2_At2g28690 RsaI
toPt-437076
pPt-651482
pPt-538862
pPt-654243
pPt-457853
pPt-653695
pPt-536043
pPt-538893
pPt-656571
pPt-535476
pPt-652099
pPt-650932
pPt-538487
capPt-674558
pPt-657042
pPt-654223
pPt-651002
pPt-536287
pPt-459522
pPt-652026
pPt-656951
pPt-654718
pPt-538271
pPt-540399
pPt-538641
pPt-458998
toPt-440725
pPt-533675
pPt-458509
pPt-537512
pPt-536575
pPt-655963
pPt-535136
pPt-538223
pPt-537893
pPt-538181
pPt-652532
pPt-539898
pPt-655155
pPt-534049
pPt-534156
toPt-437430
pPt-651297
toPt-438445
pPt-654095
pPt-537222
pPt-536274
pPt-537593
pPt-537348
pPt-653463
pPt-459517
pPt-472744
pPt-535542
pPt-535871
VI [2]
toPt-437131
pPt-655850
pPt-655265
toPt-438314
pPt-653838
pPt-473778
pPt-655768
toPt-439154
pPt-656749
pPt-538939
toPt-439178
pPt-656983
pPt-552435
pPt-535780
pPt-473555
pPt-458431
pPt-539320
pPt-539974
pPt-650403
pPt-656787
pPt-653845
pPt-552021
pPt-651023
pPt-652784
pPt-534969
pPt-657350
pPt-654722
pPt-471936
pPt-656917
pPt-650333
pPt-651401
pPt-650866
pPt-653906
pPt-539030
pPt-457662
pPt-539812
pPt-458247
pPt-471000
pPt-559151
pPt-559109
pPt-656143
pPt-654866
pPt-535435
pPt-536409
pPt-540266
pPt-538029
pPt-535842
0
5
10
15
20
25
30
35
40
45
50
55
60
65
70
75
80
85
90
95
100

## Slide 5
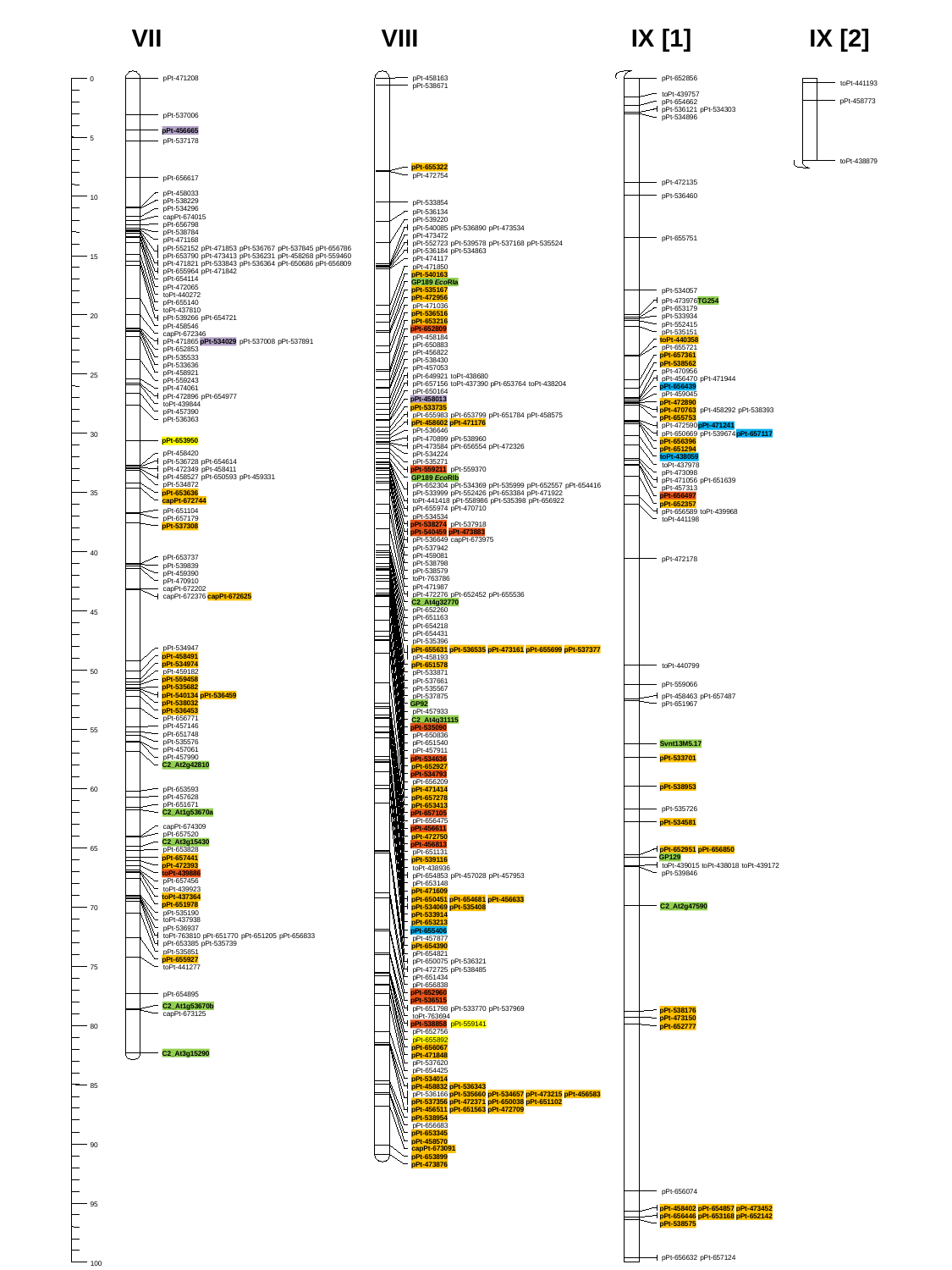

VII
pPt-471208
pPt-537006
pPt-456665
pPt-537178
pPt-656617
pPt-458033
pPt-538229
pPt-534296
capPt-674015
pPt-656798
pPt-538784
pPt-471168
pPt-552152
pPt-471853
pPt-536767
pPt-537845
pPt-656786
pPt-653790
pPt-473413
pPt-536231
pPt-458268
pPt-559460
pPt-471821
pPt-533843
pPt-536364
pPt-650686
pPt-656809
pPt-655964
pPt-471842
pPt-654114
pPt-472065
toPt-440272
pPt-655140
toPt-437810
pPt-539266
pPt-654721
pPt-458546
capPt-672346
pPt-471865
pPt-534029
pPt-537008
pPt-537891
pPt-652853
pPt-535533
pPt-533636
pPt-458921
pPt-559243
pPt-474061
pPt-472896
pPt-654977
toPt-439844
pPt-457390
pPt-536363
pPt-653950
pPt-458420
pPt-536728
pPt-654614
pPt-472349
pPt-458411
pPt-458527
pPt-650593
pPt-459331
pPt-534872
pPt-653636
capPt-672744
pPt-651104
pPt-657179
pPt-537308
pPt-653737
pPt-539839
pPt-459390
pPt-470910
capPt-672202
capPt-672376
capPt-672625
pPt-534947
pPt-458491
pPt-534974
pPt-459182
pPt-559458
pPt-535682
pPt-540134
pPt-536459
pPt-538032
pPt-536453
pPt-656771
pPt-457146
pPt-651748
pPt-535576
pPt-457061
pPt-457990
C2_At2g42810
pPt-653593
pPt-457628
pPt-651671
C2_At1g53670a
capPt-674309
pPt-657520
C2_At3g15430
pPt-653828
pPt-657441
pPt-472393
toPt-439886
pPt-657456
toPt-439923
toPt-437364
pPt-651978
pPt-535190
toPt-437938
pPt-536937
toPt-763810
pPt-651770
pPt-651205
pPt-656833
pPt-653385
pPt-535739
pPt-535851
pPt-655927
toPt-441277
pPt-654895
C2_At1g53670b
capPt-673125
C2_At3g15290
VIII
pPt-458163
pPt-538671
pPt-655322
pPt-472754
pPt-533854
pPt-536134
pPt-539220
pPt-540085
pPt-536890
pPt-473534
pPt-473472
pPt-552723
pPt-539578
pPt-537168
pPt-535524
pPt-536184
pPt-534863
pPt-474117
pPt-471850
pPt-540163
GP189 EcoRIa
pPt-535167
pPt-472956
pPt-471036
pPt-536516
pPt-653216
pPt-652809
pPt-458184
pPt-650883
pPt-456822
pPt-538430
pPt-457053
pPt-649921
toPt-438680
pPt-657156
toPt-437390
pPt-653764
toPt-438204
pPt-650164
pPt-458013
pPt-533735
pPt-655983
pPt-653799
pPt-651784
pPt-458575
pPt-458602
pPt-471176
pPt-536646
pPt-470899
pPt-538960
pPt-473584
pPt-656554
pPt-472326
pPt-534224
pPt-535271
pPt-559211
pPt-559370
GP189 EcoRIb
pPt-652304
pPt-534369
pPt-535999
pPt-652557
pPt-654416
pPt-533999
pPt-552426
pPt-653384
pPt-471922
toPt-441418
pPt-558986
pPt-535398
pPt-656922
pPt-655974
pPt-470710
pPt-534534
pPt-538274
pPt-537918
pPt-540459
pPt-473883
pPt-536649
capPt-673975
pPt-537942
pPt-459081
pPt-538798
pPt-538579
toPt-763786
pPt-471987
pPt-472276
pPt-652452
pPt-655536
C2_At4g32770
pPt-652260
pPt-651163
pPt-654218
pPt-654431
pPt-535396
pPt-655631
pPt-536535
pPt-473161
pPt-655699
pPt-537377
pPt-458193
pPt-651578
pPt-533871
pPt-537661
pPt-535567
pPt-537875
GP92
pPt-457933
C2_At4g31115
pPt-535090
pPt-650836
pPt-651540
pPt-457911
pPt-534636
pPt-652927
pPt-534793
pPt-656209
pPt-471414
pPt-657278
pPt-653413
pPt-657105
pPt-656475
pPt-456611
pPt-472750
pPt-456813
pPt-651131
pPt-539116
toPt-438936
pPt-654853
pPt-457028
pPt-457953
pPt-653148
pPt-471609
pPt-650451
pPt-654681
pPt-456633
pPt-534069
pPt-535408
pPt-533914
pPt-653213
pPt-655406
pPt-457877
pPt-654390
pPt-654821
pPt-650075
pPt-536321
pPt-472725
pPt-538485
pPt-651434
pPt-656838
pPt-652960
pPt-536515
pPt-651798
pPt-533770
pPt-537969
toPt-763694
pPt-538858
pPt-559141
pPt-652756
pPt-655892
pPt-656067
pPt-471848
pPt-537620
pPt-654425
pPt-534014
pPt-458832
pPt-536343
pPt-536166
pPt-535660
pPt-534657
pPt-473215
pPt-456583
pPt-537356
pPt-472371
pPt-650038
pPt-651102
pPt-456511
pPt-651563
pPt-472709
pPt-538954
pPt-656683
pPt-653345
pPt-458570
capPt-673091
pPt-653899
pPt-473876
IX [1]
pPt-652856
toPt-439757
pPt-654662
pPt-536121
pPt-534303
pPt-534896
pPt-472135
pPt-536460
pPt-655751
pPt-534057
pPt-473976
TG254
pPt-653179
pPt-533934
pPt-552415
pPt-535151
toPt-440358
pPt-655721
pPt-657361
pPt-538562
pPt-470956
pPt-456470
pPt-471944
pPt-656439
pPt-459045
pPt-472890
pPt-470763
pPt-458292
pPt-538393
pPt-655753
pPt-472590
pPt-471241
pPt-650669
pPt-539674
pPt-657117
pPt-656396
pPt-651294
toPt-438059
toPt-437978
pPt-473098
pPt-471056
pPt-651639
pPt-457313
pPt-656497
pPt-652357
pPt-656589
toPt-439968
toPt-441198
pPt-472178
toPt-440799
pPt-559066
pPt-458463
pPt-657487
pPt-651967
Svnt13M5.17
pPt-533701
pPt-538953
pPt-535726
pPt-534581
pPt-652951
pPt-656850
GP129
toPt-439015
toPt-438018
toPt-439172
pPt-539846
C2_At2g47590
pPt-538176
pPt-473150
pPt-652777
pPt-656074
pPt-458402
pPt-654857
pPt-473452
pPt-656446
pPt-653168
pPt-652142
pPt-538575
pPt-656632
pPt-657124
IX [2]
toPt-441193
pPt-458773
toPt-438879
0
5
10
15
20
25
30
35
40
45
50
55
60
65
70
75
80
85
90
95
100

## Slide 6
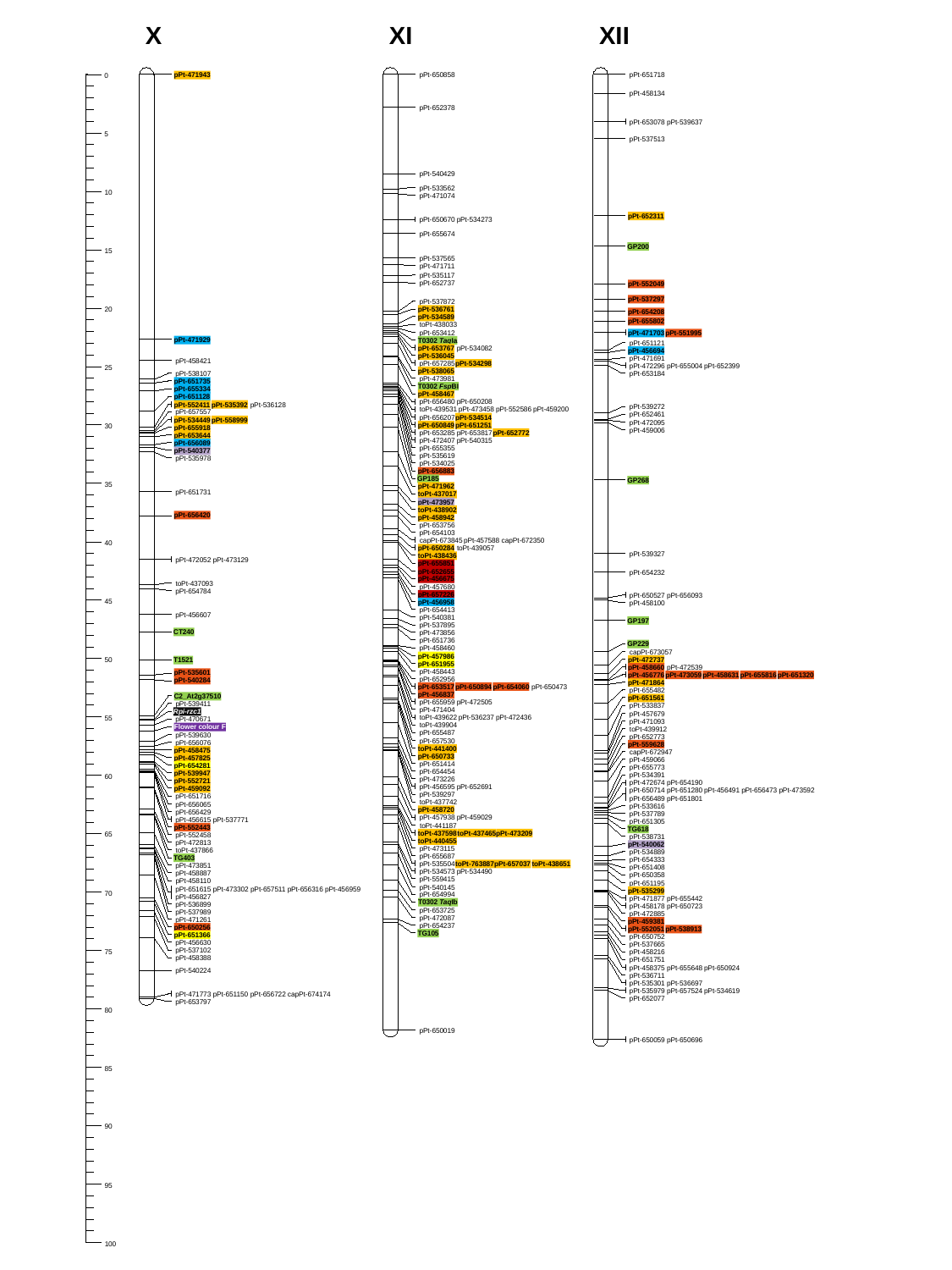

X
pPt-471943
pPt-471929
pPt-458421
pPt-538107
pPt-651735
pPt-655334
pPt-651128
pPt-552411
pPt-535392
pPt-536128
pPt-657557
pPt-534449
pPt-558999
pPt-655918
pPt-653644
pPt-656089
pPt-540377
pPt-535978
pPt-651731
pPt-656420
pPt-472052
pPt-473129
toPt-437093
pPt-654784
pPt-456607
CT240
T1521
pPt-535601
pPt-540284
C2_At2g37510
pPt-539411
Rpi-rzc1
pPt-470671
Flower colour F
pPt-539630
pPt-656076
pPt-458475
pPt-457825
pPt-654281
pPt-539947
pPt-552721
pPt-459092
pPt-651716
pPt-656065
pPt-656429
pPt-456615
pPt-537771
pPt-552443
pPt-552458
pPt-472813
toPt-437866
TG403
pPt-473851
pPt-458887
pPt-458110
pPt-651615
pPt-473302
pPt-657511
pPt-656316
pPt-456959
pPt-456827
pPt-536899
pPt-537989
pPt-471261
pPt-650256
pPt-651366
pPt-456630
pPt-537102
pPt-458388
pPt-540224
pPt-471773
pPt-651150
pPt-656722
capPt-674174
pPt-653797
XI
pPt-650858
pPt-652378
pPt-540429
pPt-533562
pPt-471074
pPt-650670
pPt-534273
pPt-655674
pPt-537565
pPt-471711
pPt-535117
pPt-652737
pPt-537872
pPt-536761
pPt-534589
toPt-438033
pPt-653412
T0302 TaqIa
pPt-653767
pPt-534082
pPt-536045
pPt-657285
pPt-534298
pPt-538065
pPt-473981
T0302 FspBI
pPt-458467
pPt-656480
pPt-650208
toPt-439531
pPt-473458
pPt-552586
pPt-459200
pPt-656207
pPt-534514
pPt-650849
pPt-651251
pPt-653285
pPt-653817
pPt-652772
pPt-472407
pPt-540315
pPt-655355
pPt-535619
pPt-534025
pPt-656883
GP185
pPt-471962
toPt-437017
pPt-473957
toPt-438902
pPt-458942
pPt-653756
pPt-654103
capPt-673845
pPt-457588
capPt-672350
pPt-650284
toPt-439057
toPt-438436
pPt-655851
pPt-652655
pPt-456675
pPt-457680
pPt-657226
pPt-456958
pPt-654413
pPt-540381
pPt-537895
pPt-473856
pPt-651736
pPt-458460
pPt-457986
pPt-651955
pPt-458443
pPt-652956
pPt-653517
pPt-650894
pPt-654060
pPt-650473
pPt-456837
pPt-655959
pPt-472505
pPt-471404
toPt-439622
pPt-536237
pPt-472436
toPt-439904
pPt-655487
pPt-657530
toPt-441400
pPt-650733
pPt-651414
pPt-654454
pPt-473226
pPt-456595
pPt-652691
pPt-539297
toPt-437742
pPt-458720
pPt-457938
pPt-459029
toPt-441187
toPt-437598
toPt-437465
pPt-473209
toPt-440455
pPt-473115
pPt-655687
pPt-535504
toPt-763887
pPt-657037
toPt-438651
pPt-534573
pPt-534490
pPt-559415
pPt-540145
pPt-654994
T0302 TaqIb
pPt-653725
pPt-472087
pPt-654237
TG105
pPt-650019
XII
pPt-651718
pPt-458134
pPt-653078
pPt-539637
pPt-537513
pPt-652311
GP200
pPt-552049
pPt-537297
pPt-654208
pPt-655802
pPt-471703
pPt-551995
pPt-651121
pPt-456694
pPt-471691
pPt-472296
pPt-655004
pPt-652399
pPt-653184
pPt-539272
pPt-652461
pPt-472095
pPt-459006
GP268
pPt-539327
pPt-654232
pPt-650527
pPt-656093
pPt-458100
GP197
GP229
capPt-673057
pPt-472737
pPt-458660
pPt-472539
pPt-456776
pPt-473059
pPt-458631
pPt-655816
pPt-651320
pPt-471864
pPt-655482
pPt-651561
pPt-533837
pPt-457679
pPt-471093
toPt-439912
pPt-652773
pPt-559628
capPt-672947
pPt-459066
pPt-655773
pPt-534391
pPt-472674
pPt-654190
pPt-650714
pPt-651280
pPt-456491
pPt-656473
pPt-473592
pPt-656489
pPt-651801
pPt-533616
pPt-537789
pPt-651305
TG618
pPt-538731
pPt-540062
pPt-534889
pPt-654333
pPt-651408
pPt-650358
pPt-651195
pPt-535299
pPt-471877
pPt-655442
pPt-458178
pPt-650723
pPt-472885
pPt-459381
pPt-552051
pPt-538913
pPt-650752
pPt-537665
pPt-458216
pPt-651751
pPt-458375
pPt-655648
pPt-650924
pPt-536711
pPt-535301
pPt-536697
pPt-535979
pPt-657524
pPt-534619
pPt-652077
pPt-650059
pPt-650696
0
5
10
15
20
25
30
35
40
45
50
55
60
65
70
75
80
85
90
95
100
